# Morphological and chemical changes in Cd-free colloidal QD-LEDs during operation

**DOI:** 10.1126/sciadv.aec8208

**Published:** 2026-07-10

**Authors:** Ruiqi Zhang, Jamie Geng, Shaun Tan, Mike Dillender, Shreyas Srinivasan, Taehyung Kim, Mayuran Saravanapavanantham, Kwang-Hee Kim, Heejae Chung, Sujin Park, Thienan Nguyen, Karen Yang, Yongli Lu, Tae-Gon Kim, Moungi G. Bawendi, Vladimir Bulović

**Affiliations:** ^1^ Department of Electrical Engineering and Computer Science, Massachusetts Institute of Technology, Cambridge, MA 02139, USA.; ^2^ Research Laboratory of Electronics (RLE), Massachusetts Institute of Technology, Cambridge, MA 02139, USA.; ^3^ Department of Chemistry, Massachusetts Institute of Technology, Cambridge, MA 02139, USA.; ^4^ Samsung Advanced Institute of Technology, Samsung Electronics, Suwon, Republic of Korea.

## Abstract

Heavy-metal-free quantum-dot light-emitting devices (QD-LEDs) demonstrate high brightness, saturated color, and high efficiency, yet their operational lifetimes remain limited, with the underlying degradation mechanisms not fully understood. Here, we show that InP/ZnSe/ZnS (red-emitting) and ZnTeSe/ZnSe/ZnS (blue-emitting) colloidal QD-LEDs undergo nanoscale morphological changes during operation. Interparticle coarsening and layer thinning are observed in the core functional layers, accompanied by the generation and diffusion of compositional-oxygen and hydrogen across the device, with oxygen accumulating at the Al electrode/ZnMgO electron-transport layer (ETL) interface. In situ transmission electron microscopy reveals that electron beam exposure, in presence of atomic hydrogen species, accelerates ZnMgO nanoparticles coarsening. To mitigate these degradation pathways, we show that acrylate-based resin encapsulation can stabilize the ETL, HTL, and QD layers by suppressing atomic species formation and halting morphology changes. This approach achieves over 50-fold and 5000-fold lifetime improvement in InP/ZnSe/ZnS and ZnTeSe/ZnSe/ZnS QD-LEDs, respectively. Our findings establish the causal relationships between morphological degradation, interlayer dynamics, and QD-LED instability, providing insight into the acrylate encapsulation treatment that enables efficient and long-lived QD-LEDs.

## Teaser

Resin encapsulation prevents atomic species-driven QD-LEDs morphology change, improving device efficiency and operating lifetime.

## INTRODUCTION

Colloidal quantum dot light emitting diodes (QD-LEDs) are an emerging technology with potential to be used in the next generation of high-brightness displays with saturated color, and wide-color gamut (*1*–*4*). Within last decades, QDs have been used in display technologies as optical downconverters in liquid crystal displays and in organic LED displays. Developments of electrically excited QDs in QD-LEDs have demonstrated a light-emitting technology that is compatible with flexible substrates (*5*), is environmentally benign (*6*), has fast response times (*7*), and generates efficient light emission from a simpler device architectures that lead to lower display manufacturing costs (*8*). State-of-the-art red and green QD-LEDs have demonstrated high external quantum efficiency (EQE), approaching the light out-coupling theoretical limit (*9*–*12*). EQE of blue QD-LEDs has also been improved with recent approaches, such as suppressing organic-inorganic interfacial charge leakage (*13*), and surface engineering (*14*). However, for QD-LEDs to be commercially viable, improvements must be made to their operating lifetimes, while maintaining high efficiency, especially for heavy-metal-free QD-LEDs.

QD photoluminescence (PL) efficiency, and balanced charge injection of electrons and holes into the QD layer, are the dominating factors influencing QD-LED EQE. It is found that continuously applying a forward bias to the Cd-free QD-LEDs has multiple drawbacks, including the presence of quantum-confined Stark effect that quenches the device PL, deteriorated EQE from excessively charged QDs which enhances Auger recombination (*15*), charge accumulation in transport layers, nonradiative recombination center formations (*2*, *16*, *17*), retarded carrier-induced electron injection barriers against emission layer (EML) (*9*, *18*, *19*), and the junction space-charge accumulation (*19*–*22*). Thus, to facilitate charge injection and carrier balance, metal (e.g., Mg and Al)–doped ZnO nanoparticles (NPs) are commonly selected as electron transport layer (ETL) material, modulating the interlayer band mismatch by raising the conduction band minimum. This has been shown to increase electron injection rate, suppress excessive electron flux, and diminish exciton quenching (*23*–*25*). So far, over 30% QD-LED EQE has been achieved (*26*), with high-efficiency QD-LEDs benefiting from the shelf-aging phenomenon (*27*, *28*). It has been shown that QD-LEDs containing the InP/ZnSe/ZnS red QD-emissive layer, with ZnMgO ETL, exhibit aging over weeks with an initially increasing EQE (positive aging) followed by an extended decrease in EQE (negative aging) (*29*). With latest approaches introducing water and resin encapsulation treatments, ZnMgO NPs are found to be morphologically stable and retain their n-doping, while further improving the QD-LED performance (*27*, *30*–*33*). However, the QD EML still suffers from electric field inhomogeneity, causing device degradations (*34*).

As the operational EQE degradation deteriorates an initially efficient QD-LED, this suggests that under applied electric field, QD-LEDs could undergo chemical or morphological modifications that affect QD PL and the injected charge balance. In the present study, we systematically investigate the potential device degradation mechanisms under forward-bias. We investigate the nanoscale morphological changes in the cross-sectional structures of pristine and aged red and blue Cd-free QD-LEDs composed of InP/ZnSe/ZnS and ZnTeSe/ZnSe/ZnS (core/shell/shell) QDs, respectively. We observe that progressive device aging induces densification across all functional layers of a QD-LED, resulting in layer thinning and structural rearrangements that correlate with device efficiency loss. By tracing the atomic composition of each QD-LED layer, we identify elemental motility under device bias. To assess the changes in the chemical composition of QD-LED layers as they undergo morphological changes, in situ environmental transmission electron microscopy (TEM) on drop-casted ZnMgO NPs thin films are performed, as well as on blue QD-LED lamella cross sections under hydrogen doping. We show that the presence of atomic hydrogen species accelerates coarsening of ZnMgO NPs both in the neat films, and within the ETL of blue QD-LED cross sections. In contrast, when acrylate-based resin is applied as an encapsulation treatment, both red and blue QD-LEDs maintain a stabilized ETL/QD EML morphology, and it appears that atomic species formation is suppressed, diminishing interparticle coarsening and layer ripening. The acrylate-based resin encapsulation treatment leads to a large operational lifetime improvement in both red and blue QD-LEDs.

## RESULTS

### Cd-free QD-LEDs synthesis and electro-optical properties

**Fig. 1.**
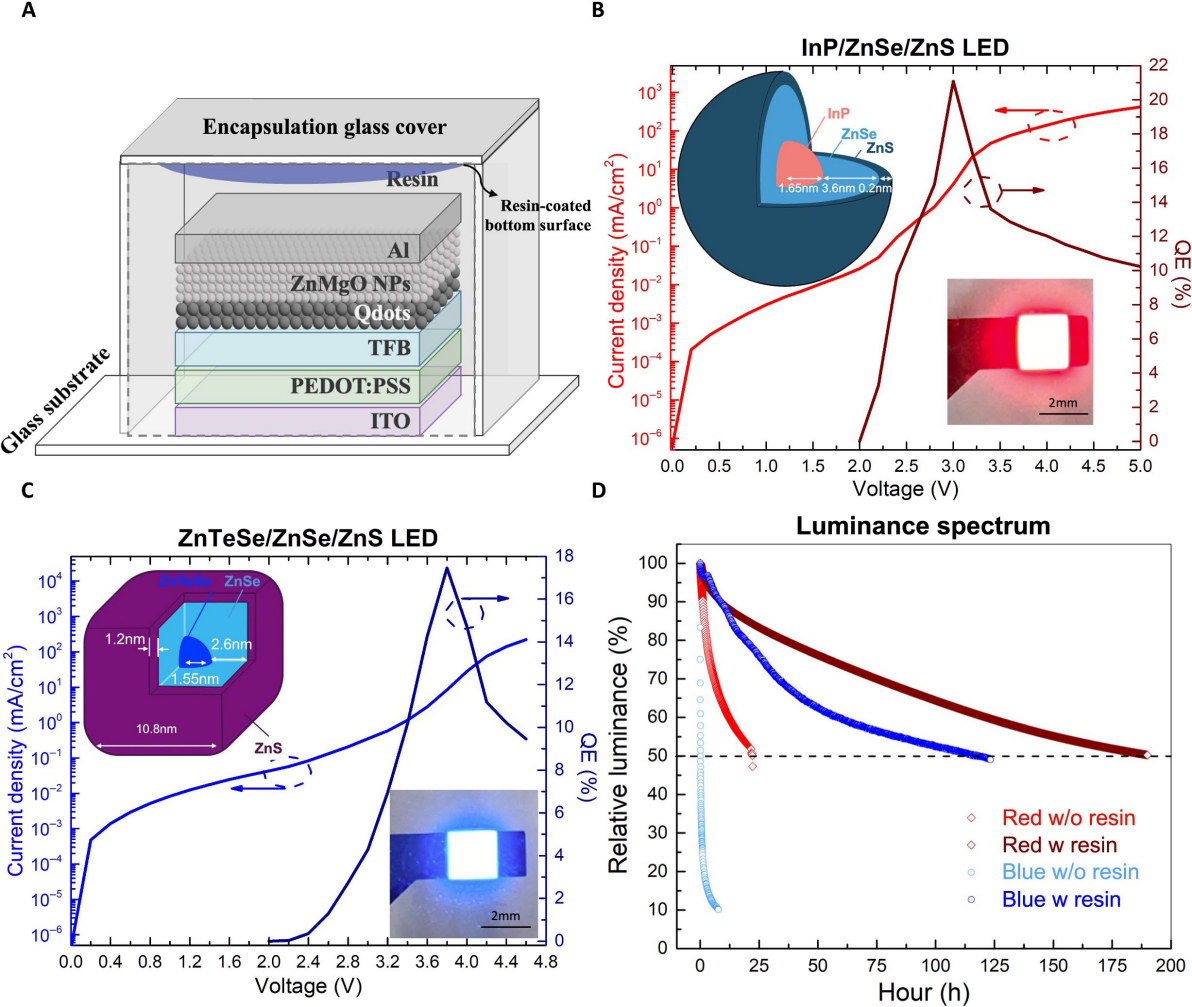
QD-LEDs structure and properties. (**A**) Resin-encapsulated Cd-free QD-LED structures, consisting of ITO/PEDOT:PSS/TFB/QDs/ZnMgO NPs/Al/remote resin/glass encapsulation. (**B** and **C**) Operational electronic properties of (B) InP/ZnSe/ZnS resin-encapsulated QD-LEDs and (**C**) ZnTeSe/ZnSe/ZnS resin-encapsulated QD-LEDs. For each plot, current-voltage response of QD-LEDs under forward bias is shown on the left *y* axis, where current density is plotted on the log scale (light red and light blue). Right *y* axis indicates the % EQE (dark red and dark blue) assuming the QD-LED is a Lambertian emitter. Inserted pictures show the functional devices and include the sketches of constituent QD core/shell/shell (C/S/S) structures with radii lengths indicated. Red QD C/S/S structure consists of InP, ZnSe, and ZnS with radii of 1.65, 3.6, and 0.2 nm, respectively. Blue QD C/S/S structure consists of ZnTeSe, ZnSe, and ZnS with radii of 1.55, 2.6, and 1.2 nm, respectively. Resin-encapsulated, resin-free red QD-LEDs and resin-free blue QD-LEDs have a device area of 4 mm^2^ (2 × 2 mm^2^). Resin-encapsulated blue QD-LEDs have a device area of 2 mm^2^ (1 × 2 mm^2^). (**D**) Influence of acrylate-based resin encapsulation on QD-LED operation lifetime, showing lifetime enhancement.

Figure 1A and fig. S1 illustrate the typical Cd-free QD-LED consisting of indium tin oxide (ITO)/poly(3,4-ethylenedioxythiophene):poly(styrenesulfonate) (PEDOT:PSS) [hole injection layer (HIL)]/poly(9,9-dioctylfluorene-alt-*N*-(4-sec-butylphenyl)-diphenylamine) (TFB) [hole transport layer (HTL)]/QDs light EML/ZnMgO NPs (ETL)/Al, with EML composed of InP/ZnSe/ZnS QDs (referred to in the text below as InP QDs) and ZnTeSe/ZnSe/ZnS QDs (referred to as ZnTeSe QDs), respectively. Followed by the core-shell consecutive precursor nucleation and purification procedure (*3*, *9*), InP QD cores (*r* = 1.65 nm) are formed to be uniformly spherical and covered with tailored ZnSe (*r* = 3.6 nm) and ZnS (*r* = 0.2 nm) shells, suppressing nonradiative Auger recombination. ZnTeSe-based QDs tend to form quasi-cubic/diamond shapes, with a ZnTeSe core, ZnSe inner shell, and ZnS outer shell with 1.55m, 2.6, and 1.2 nm radii lengths, respectively (Fig. 1, B and C, insets). Emission wavelength of the Te-doped ZnSe QD core in blue QD-LEDs is tuned by adjusting the Te/Se composition ratio, with an addition of hydrofluoric acid (HF) exposure to suppress the stacking faults during the QD shell growth process (*9*). Application of forward bias leads to QD-LED electroluminescence (EL), with InP QD-LEDs turning on at around 2.0 V, with a peak EL emission wavelength at λ = 630 nm [1.97 eV peak energy, full width at half maximum (FWHM) of 110 meV]. ZnTeSe QD-LEDs turn on at around 2.4 V, with a peak EL emission wavelength at λ = 465 nm (2.67 eV peak energy, FWHM of 250 meV).

To investigate device aging, the fabricated red and blue QD-LEDs are subjected to an applied bias, adjusted to maintain a constant device current. The steady current is maintained until the device brightness decreases to 50% of its initial value, at the time referred to as LT50 lifetime. Resin-free InP QD-LEDs have an LT50 of 22.1 hours (with initial condition set as 3.35 V, 58.8 mA/cm^2^, generating 2070 cd/m^2^ device brightness). Resin-free ZnTeSe QD-LEDs have an LT50 of 0.2 hours (when initiated at 3.35 V, 2.5 mA/cm^2^, and 146 cd/m^2^).

To enhance the device performance, remote acrylate-based resin encapsulation treatment is applied, leading to an extended T50 lifetime in both InP and ZnTeSe QD-LEDs. Resin layer is spun cast on the encapsulation glass, positioned to face the QD-LED, and not in direct contact with any QD-LED layers (fig. S1C). Resin-encapsulated QD-LEDs show extended operating lifetimes, with InP QD-LED LT50 rising to 189.9 hours (when initiated at 3.06 V, 50 mA/cm^2^, 6500 cd/m^2^) and ZnTeSe QD-LED LT50 rising to 115.5 hours (when initiated at 3.56 V, 3.9 mA/cm^2^, and 500 cd/m^2^). Assuming an accumulation factor of 1.8 (*9*), the resin-encapsulated InP QD-LED is expected to have an over 50-fold increase in T50 lifetime as compared to the resin-free device, if operated at 100 cd/m^2^. Similarly, resin-encapsulated ZnTeSe QD-LED is expected to have an over 5000-fold increase in T50 lifetime as compared to resin-free device, if operated at 100 cd/m^2^. The peak EQE of representative acrylate-based, resin-encapsulated red and blue QD-LEDs is measured as 21.1 and 17.5%, respectively, assuming Lambertian QD-LED emission (*35*) (see Fig. 1). A statistical result of device performances is further presented in figs. S2 and S3. Luminance spectra are shown in fig. S4.

### Device nanomorphology variations and interlayer elemental tracing

**Fig. 2.**
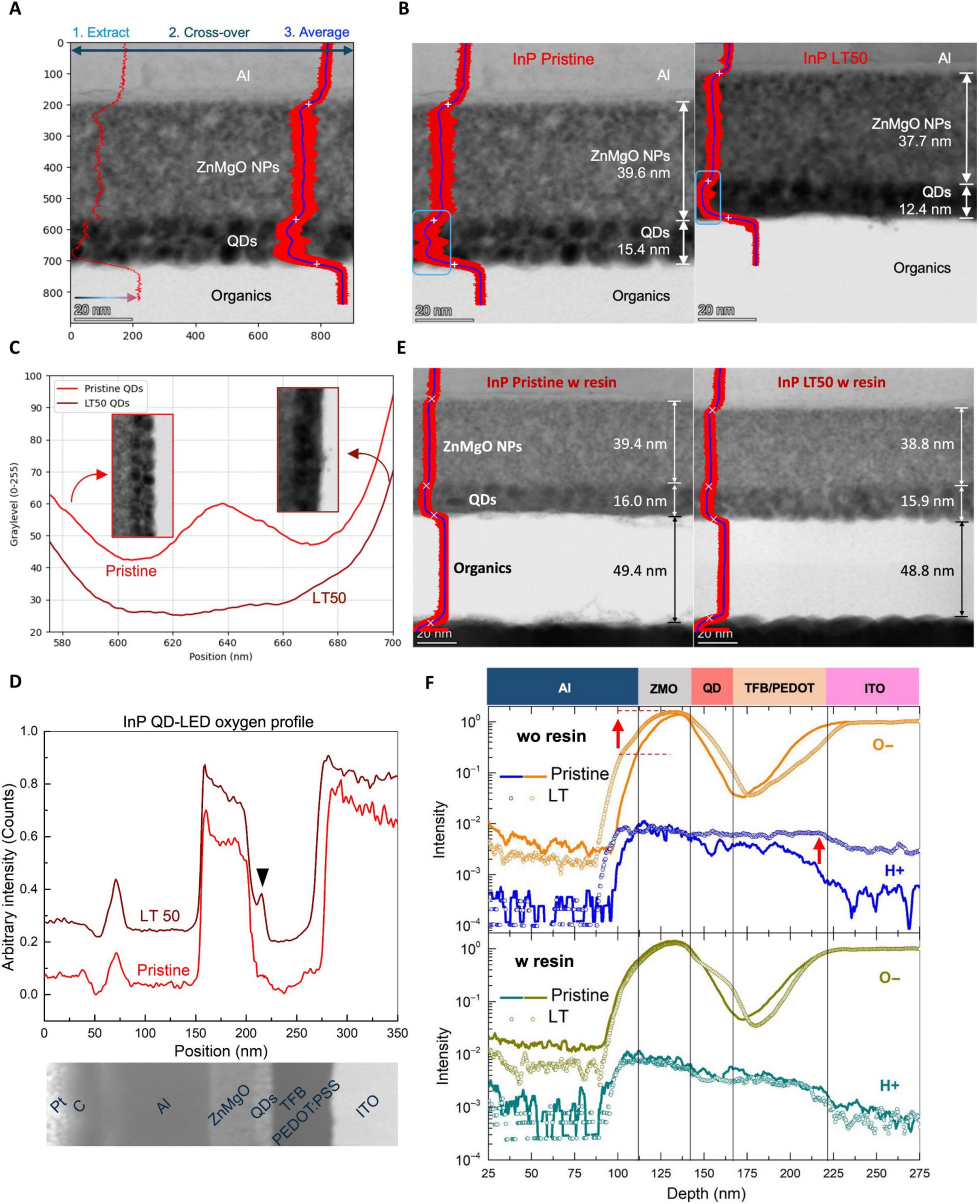
Degradation of InP QD-LEDs with and without acrylate-based resin. (**A**) Quantitative analysis of a QD-LED cross-sectional TEM image. Brightness of the first column of pixels is plotted as the red line on the left. Brightness of all the columns of pixels are overlapped on the right, with a dark-blue line indicating the average response. Midpoints of the slopes for each extracted column are averaged and indicated as white cross-markers at vertical-axis index around 200, 580, and 700 in the image. (**B**) Cross-sectional TEM image of pristine (left) and LT50-aged (right) InP/ZnSe/ZnS QD-LED. (**C**) Grayscale level of the zoomed in InP/ZnSe/ZnS QD layer versus depth position spectrum. The QD grayscale brightness is obtained from the image regions that are marked with light blue rectangles in (B). (**D**) EDS oxygen spectrum of red InP QD-LED with its cross-sectional TEM image inserted below. Pristine and LT-aged curves are plotted in light and dark red color, correspondingly. An extra oxygen peak is observed in InP/ZnSe/ZnS QD layer after aging, indicating the operationally induced oxygen migration. At the Al/ZnMgO junction, the spectrum indicates an increase in the oxygen level in both pristine and aged red QD-LEDs. (**E**) Cross-sectional TEM image of pristine (left) and LT50-aged (right) acrylate-based, resin-encapsulated InP QD-LEDs. (**F**) TOF-SIMS spectrum of O^−^ and H^+^ species in pristine and LT50-aged InP QD-LEDs. Lower and upper spectra correspond to the QD-LED with and without acrylate-resin encapsulation, respectively. QD-LED layer information is indicated on top.

To examine the degradation processes of devices with and without resin encapsulation, we measure the nanoscale morphology and chemistry of a set of cross-sectioned QD-LEDs. Focused ion beam (FIB) is used to prepare lamellas of pristine red and blue QD-LED, T50-aged red QD-LED and T70-aged blue QD-LED, with lamella preparation process shown in fig. S5. Lamella thickness is controlled to be less than 200 nm to maximize collected transmission electron microscopy (TEM) signal-to-noise level. To measure the QD-LED layer thickness from TEM images, we develop an image analysis algorithm, shown in Fig. 2A (also the Supplementary Materials Demo Code). A raw TEM image is extracted into column vectors with each vector representing a column-wise grayscale level, ranging from 0 to 255 (red line on the left of Fig. 2A shows the brightness of the first column of image pixels). Column vectors are then horizontally summed and averaged to extract the pixel-wise layer-thickness curves based on the image dimension (overlapping red line on the right, with a blue line indicating the average). Centers of the rising and falling slopes of each individual column vector are calculated and averages of these center points are designated as edges of constituent layers, as shown in Fig. 2A and fig. S6. These center points determine the constituent layer thicknesses, with ±0.5 nm uncertainty, as shown in Fig. 2B.

The cross section of an aged InP QD-LED in Fig. 2B shows coarsening and compression in the QD layer as well as in the ZnMgO NPs layer. QDs in the aged device show blurred boundaries as compared to the QDs in the pristine device. A 4.8% post-aging thickness reduction in ZnMgO layer and a 19.5% decrease in QD layer thickness are observed. Notably, a clear QD bilayer is observed through the gray-level thickness contrast analysis of pristine QD-LEDs (Fig. 2C). However, this contrast disappears after QD-LED aging. Moreover, these aging-induced morphological changes unevenly shrink the core functional layer thickness, forming nonuniform junction areas and interfaces, causing imbalanced carrier injections. The observed disappearance of QD bilayer structure could be related to the previously suggested QD-ligand detachment and rearrangement under electrical bias (*36*, *37*), interfacial interaction between QD layers and the adjacent ZnMgO NPs layer (*38*, *39*), and/or an increased local heating (*40*). We would expect that these morphological variations further influence the ligand-binding conditions that lead to energy band shifts at QD interfaces with charge transport layers (*41*), QD degradation (*10*, *21*, *42*), and increased exciton quenching (*19*) in aged devices.

To probe the bias-induced interlayer elemental diffusions, spatially resolved composition mapping is collected using energy-dispersive x-ray spectroscopy (EDS). Figure 2D and fig. S7 show the oxygen EDS mapping profile across the InP QD-LED cross section for devices without resin packaging (also see fig. S8). An increase in the oxygen profile intensity at the ZnMgO/Al interface appears on both pristine and aged devices, suggesting formation of an oxidized Al layer. A new oxygen peak is also observed in the LT50-aged QD layer, suggesting that oxygen migrates across the neighboring layers into the QD layer.

In comparison, resin-encapsulated InP QD-LEDs show a stable interlayer morphology after T50 operational aging, as shown in Fig. 2E. Furthermore, the extra oxygen peak in the QD layer is not present in resin-encapsulated devices, as shown in fig. S9. It is reported that acrylic acid resin (CH_2_═CHCOOH) is able to react with hydroxyl group (─OH) inside ZnMgO that generates water, chemically absorbing and interacting with the ZnMgO surface (*27*). This water-treated ETL introduces the oxygen absorption sites, likely hindering the release of oxygen species from ZnMgO layer that could further diffuse into the QD layer.

Time-of-flight secondary ion mass spectrometry (TOF-SIMS) is carried out to investigate aging effects on the compositional species within InP QD-LEDs, as shown in fig. S10. To investigate the potential changes in the oxidation and reduction behaviors before and after device aging, Fig. 2F shows a comparison between O^−^ and H^+^ level within the device layers of InP QD-LEDs with and without the resin. Without resin-encapsulation, the normalized oxygen and hydrogen intensity levels change as the devices operate (fig. S11). With resin treatment, the oxygen level is steady in both the pristine and the LT50-aged devices throughout the ZnMgO layer and at both Al/ZnMgO and ZnMgO/QDs junctions. The change in the O^−^ concentration near the QD/HTL layers interface shows the increase in the O^−^ within the QD layer after aging, and a concomitant decrease of O^−^ within the organic HTL layer. As the aged resin-encapsulated devices do not show the EDS oxygen peak at the QD layer (fig. S9), this suggests that the EDS oxygen peak present at the QD layer in the resin-free devices is likely due to the oxygen atoms provided by the neighboring ZnMgO NP layer, as the changes in the oxygen level in the ZnMgO NP layer are inhibited when resin is present.

Moreover, H^+^ maintains a constant level after aging in resin-encapsulated devices, indicating that the presence of the resin suppresses the potential bias-induced device reduction. Note that an elevated hydrogen signal is observed at the HTL/ITO interface in the LT50-aged resin-free devices. This may arise from the field-driven migration and interfacial trapping of extra hydrogen-species observed in the device. Under electrical stress, ITO is electrochemically active where protonic species may drift from adjacent organic layers and become incorporated at the oxygen-deficient sites in the porous polycrystalline ITO (*43*, *44*). Resin encapsulation suppresses this hydrogen accumulation (also see fig. S12).

**Fig. 3.**
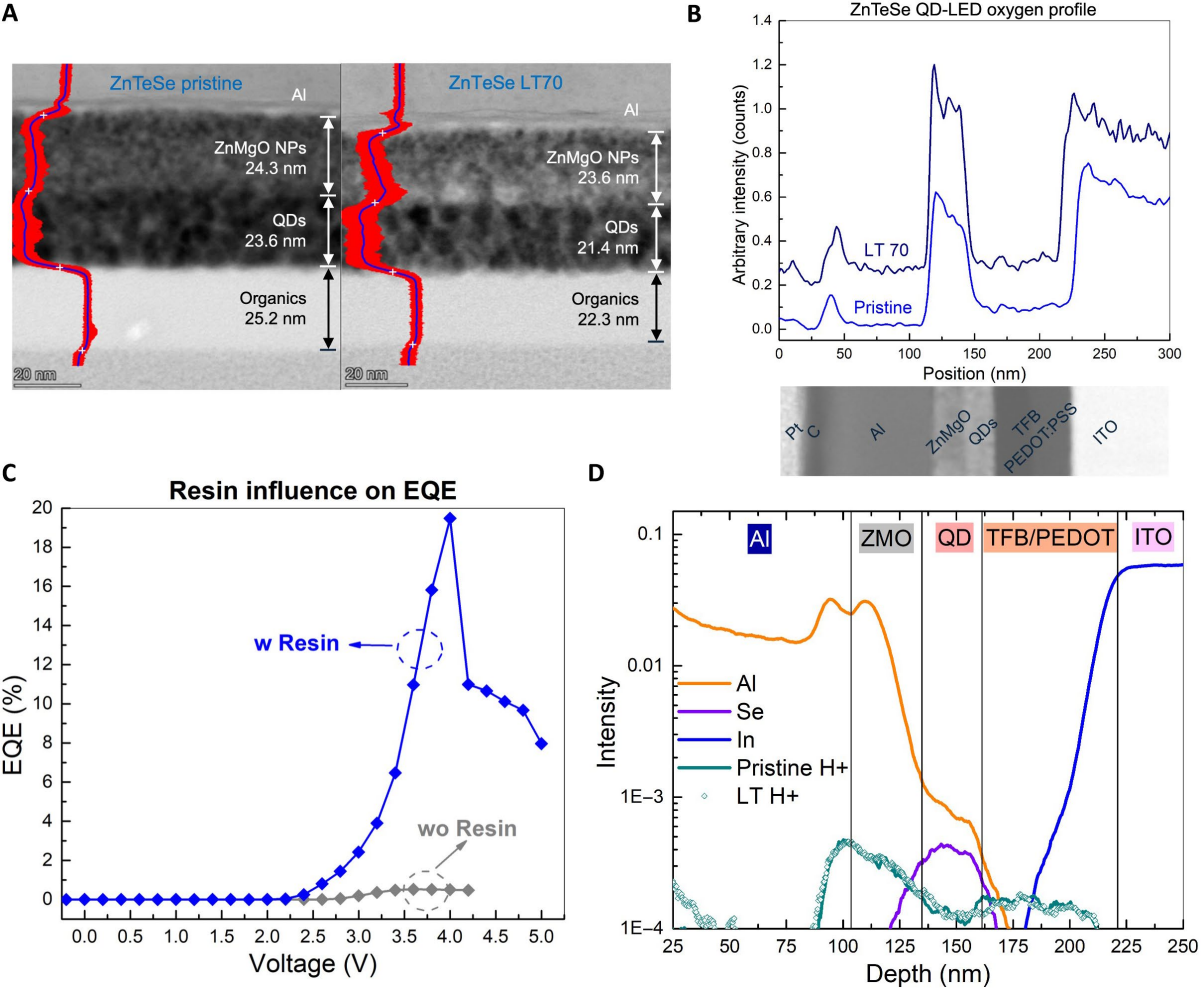
ZnTeSe QD-LED degradation characterization. (**A**) Cross-sectional TEM images of pristine and LT70-aged resin-free ZnTeSe QD-LED. Reduction of layer thickness for aged device is observed in QD, ETL, and HTL (organics) layers. (**B**) Oxygen EDS spectra of pristine and aged resin-free ZnTeSe QD-LED with a cross-sectional TEM image of the pristine device inserted below. The spectra show an increase in the oxygen level at the Al/ZnMgO junction. (**C**) Resin influence on ZnTeSe QD-LED EQE. Blue and gray curves represent the device with and without the resin, respectively. (**D**) TOF-SIMS spectrum of ZnTeSe QD-LED with resin. Solid line and scatter plot represent pristine and LT50-aged device, respectively. For devices encapsulated with resin, H^+^ level is not changed as the device is aged.

Compared with InP QD-LEDs, LT70-aged resin-free ZnTeSe QD-LEDs show a 2.9% reduction in ZnMgO layer thickness and a 9.3% reduction in QDs layer, as shown in Fig. 3A. An 11.5% thickness reduction is also observed in the organic HTL (TFB) layer. In contrast to the InP QD-LEDs, ZnTeSe QD-LEDs do not show oxidation of the QD layer, as shown in Fig. 3B (also see fig. S13). We note that the rapid EQE decay (over 0.2 hours) of the resin-free ZnTeSe QD-LED substantially reduces the total amount of charge that passes through the device, as compared to the much longer lived InP QD-LEDs, which would lead to the reduced absolute amount of oxygen motility. Similar to the resin-encapsulated InP QD-LEDs, acrylate-resin-encapsulated ZnTeSe QD-LED exhibits an elongation in the LT50 time. With the same turn-on voltage, Fig. 3C illustrates a boost in EQE for the acrylate-resin-encapsulated ZnTeSe QD-LED, consistent with the reported n-doping that increases the conductivity of ZnMgO layer, while further enhancing the device EQE. After T70 degradation, an unchanged TOF-SIMS H^+^ level further confirms the effectiveness of the resin-encapsulation in inhibiting elemental diffusions throughout the QD-LED, extending the device lifetime, as shown in Fig. 3D (TOF-SIMS spectra for resin-free blue QD-LED are shown in figs. S14 and S15).

During device operation, self-heating within the QD-LED might be another factor that contributes to morphological degradation. To further evaluate if the QD layer could be heated significantly during electrical aging process, we constructed a thermal simulation of glass-encapsulated QD-LEDs to find the steady-state temperature distribution. Lifetime measurements for the resin-encapsulated ZnTeSe-based QD-LED are recorded with a current density of 4 mA/cm^2^ and an applied voltage of 3.6 V. For the resin-encapsulated InP-based QD-LED, the lifetime measurements are recorded under 3.1 V, 50 mA/cm^2^. Figure S16 illustrates the simulation results for both blue and red encapsulated QD-LEDs under the same driving current and voltage, showing that when initiated at 293 K, the steady-state current passage increases the device surface temperature by 0.3 and 2.9 K for blue and red QD-LED, respectively. This minimal local heating during device aging is likely not the dominant cause of the observed device degradation.

### ZnMgO NPs ripening with hydrogen doping

Previous studies have shown that aging of the ZnMgO NPs layer contributes to EQE degradation of Cd-free QD-LEDs (*29*), and that this aging process is closely linked to the local chemical environment, particularly when resin encapsulated. To investigate changes on ZnMgO NP thin film morphology when exposed to different chemical environments, we measured the morphology of ZnMgO NP thin films after exposed to hydrogen and oxygen species.

ZnMgO NP thin films are prepared by drop-casting their colloidal solution onto epoxy-coated TEM grids, enabling a TEM analysis, which we perform under different in situ conditions. The electron beam generated from the field-emission gun of the high-resolution TEM (HR-TEM) induces ionization of residual O_2_ and H_2_ gases, so we first assess the effects of TEM beam irradiation on ZnMgO thin film before radical exposure.

**Fig. 4.**
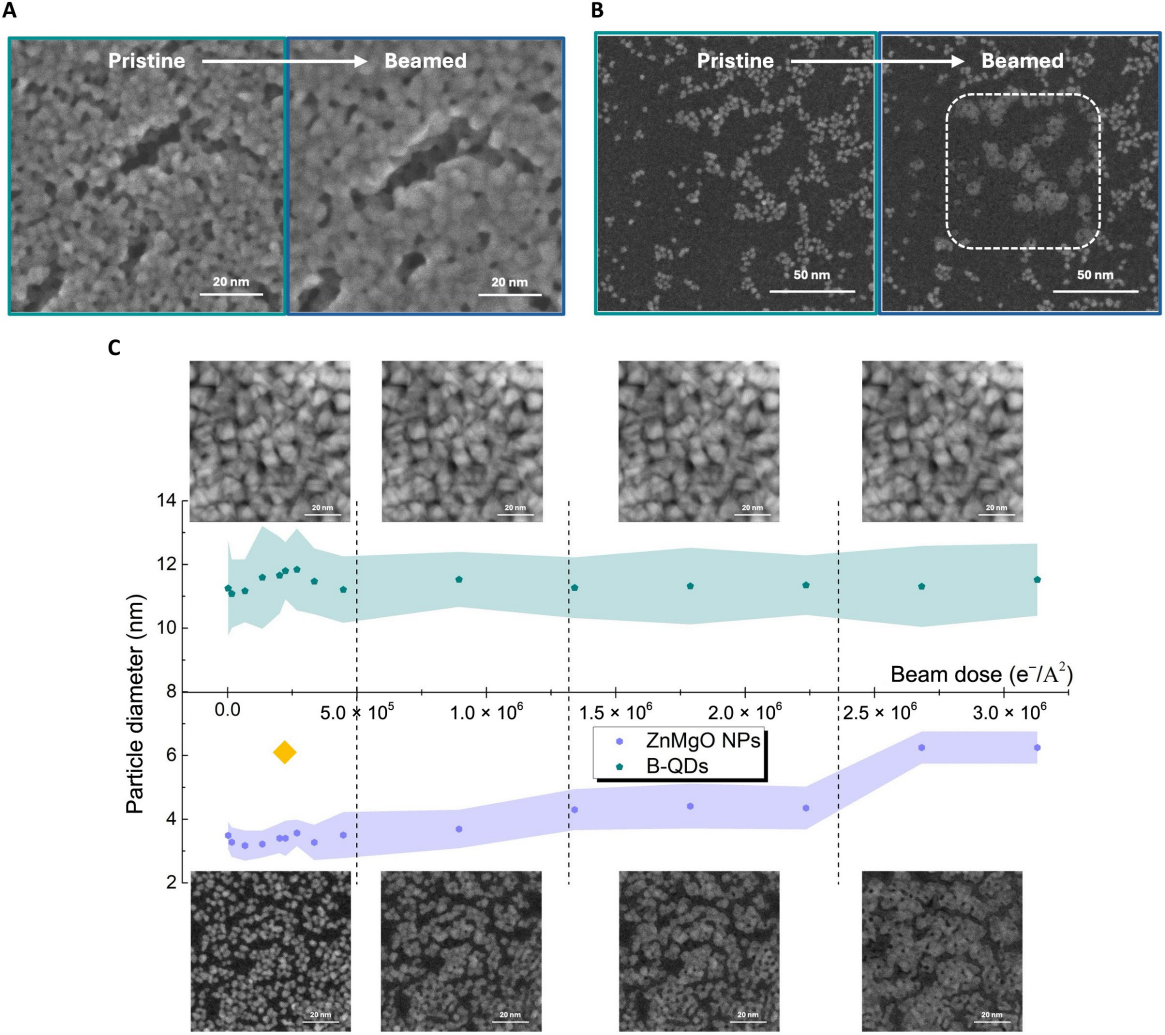
Influence of electron beam irradiation on films of ZnMgO NPs and ZnTeSe QDs. HR-TEM images of (**A**) pristine (left) and beam irradiated (right) ZnMgO NPs thin film acquired in the same region of interests. An increase in NP size and a coarsening of NP boundaries is observed. (**B**) Influence of beam irradiation on ZnMgO NPs with area of less densely packed particles. White rectangle indicates the area where electron beam was directed. Similar coarsening effect is observed as in (A) above. (**C**) Quantitative analysis of coarsened particle diameter versus total injected electron beam dose. At each dose, more than 30 randomly chosen particles are measured, from the corresponding images, to get their averaged diameter. The purple-colored band of measured values represent the scatter plot of particle diameters of ZnMgO NPs. Also shown is the cyan-colored band of measured values representing the scatter plot of particle diameters of ZnTeSe QDs, under various dozes of TEM electron beam irradiation. Solid filling of the area covering the scatter points illustrates the measured error bar at each dose density. Yellow diamond illustrates the dose at which we observed the ZnMgO NP coarsening when hydrogen is present, as further discussed in Fig. 5 below.

Pristine ZnMgO thin film is first imaged with an acquisition time of 17.6 s (corresponding to the electron irradiation dose of $1.59\times 10^{4}\;\frac{e^{-}}{{Å}^{2}}$), limiting the potential electron beam damage of the film. Electron beam is then directed to continually irradiate an area of a film for an extended period of time, injecting charge through the film. Figure 4A and fig. S17 illustrate the influence of beam irradiation on the ZnMgO NPs where after 10 min of beam exposure ($2.24\times 10^{6}\;\frac{e^{-}}{{Å}^{2}}$), ZnMgO NPs undergo coarsening into larger shapes, with less-defined particle boundaries. The same experiment is repeated on a less densely packed ZnMgO NP film. As shown in Fig. 4B, circled area has undergone a continuous beam irradiation and exhibits coarsening, with neighboring NPs merging and evolving from NP clusters into thin film textures. To quantitatively investigate the relationship between the total electron dose passing through the ZnMgO layer versus the level of coarsening, we calculate the electron beam dose density per unit area based on the TEM’s inherent current density, probe size, dose rate, acquisition window frame rate, and irradiation time (*45*–*47*). As shown in Fig. 4C, we find that as total dose increases from $2.39\times 10^{3}\;\frac{e^{-}}{{Å}^{2}}$ to $4.47\times 10^{5}\;\frac{e^{-}}{{Å}^{2}}$, no significant coarsening is observed. However, starting at a higher electron dose of $8.94\times 10^{5}\;\frac{e^{-}}{{Å}^{2}}$, ZnMgO NPs begin to aggregate and exhibit ripened coarsening. An obvious loss of NP features appears at a dose higher than $2.24\times 10^{6}\;\frac{e^{-}}{{Å}^{2}}$ (see figs. S18 and S19), demonstrating the degradation impact on ZnMgO NPs due to high-energy electron beam current passing through. In a separate experiment, a similar range of TEM electron beam doses are applied to a drop-casted ZnTeSe QDs thin film, with no obvious morphological changes observed (Fig. 4C).

**Fig. 5.**
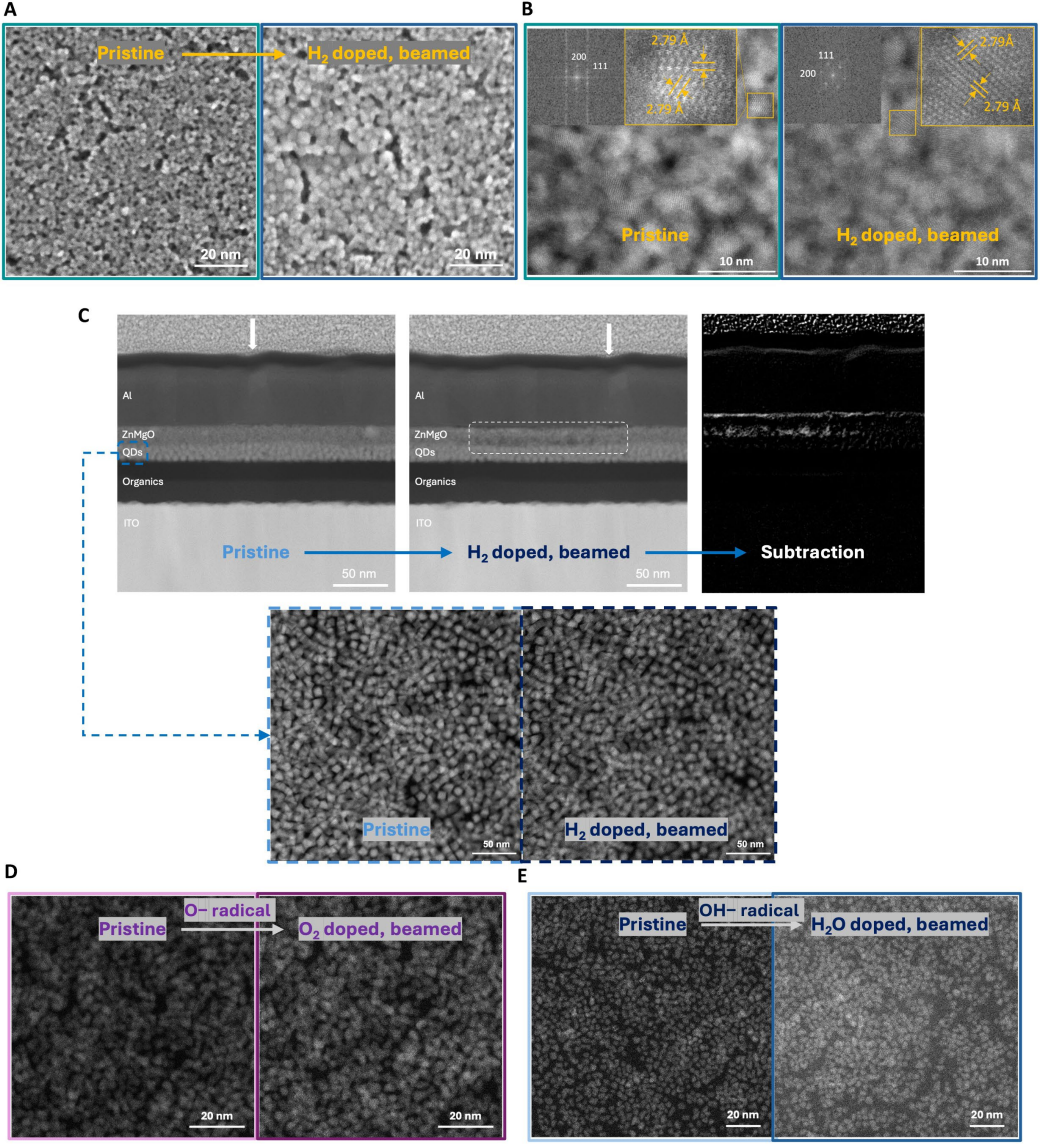
Influence of hydrogen doping, oxygen-doping, and water-doping on ZnTeSe/ZnSe/ZnS QD-LEDs. HR-TEM image of (**A**) pristine (left) and TEM-beam-irradiated and hydrogen-doped ZnMgO NPs thin film (right). Same region of interest is shown. Similar coarsening and enlarging of NPs are observed with a smaller electron dose density, as compared to the experiments with no hydrogen present. (**B**) Atomic-resolution HR-TEM images of pristine and hydrogen-doped ZnMgO NPs thin films. Inserted images (upper right) indicate the zoomed-in atomic structure of the selected region. Atomic lattice spacings are measured in both HR-TEM images. Inserted images (upper left) represent the 2D fast Fourier transform (FFT) patterns of the atomic structure extracted from the raw image. ZnO (111) and (200) planes are acquired in the in-plane direction. (**C**) TEM e-beamed and H-doped ZnTeSe QD-LED cross-sectional lamella. A coarsened ZnMgO NPs layer is observed after TEM electron beam irradiation and hydrogen doping. The white box indicates the area where extended electron beam irradiation was performed after hydrogen doping, showing the NP coarsening. White arrows indicate the same device feature on the lamella, which allows us to subtract the image before the extended TEM irradiation from the one after the irradiation. HR-TEM images of (**D**) beam-irradiated and O_2_-doped and (**E**) beam-irradiated and H_2_O-doped pristine (left) and doped ZnMgO NPs thin film (right). The same amount of electron dose is applied to ZnMgO NP films doped with O_2_ and with H_2_O, as was applied to ZnMgO NP films doped with H_2_, but no significant coarsening of NPs is observed.

As previously illustrated (Figs. 2F and 3D), both compositional hydrogen and oxygen are detected in InP and ZnTeSe QD-LEDs, regardless of resin encapsulation, indicating compositional species generation and migration within the ZnMgO ETL and in the adjacent QD emissive layer. Devices without resin treatment exhibit a pronounced increase in species concentration in the charge injection/recombination region. To assess the influence of hydrogen exposure, the drop-casted ZnMgO NPs thin film are in situ exposed to H_2_ gas during TEM imaging. H_2_ gas is introduced into the TEM chamber at 3 sccm, exposing the ZnMgO-coated TEM grid to hydrogen-rich conditions. The TEM beam is concomitantly turned on, generating hydrogen species. We observe that under the exposure to both hydrogen and the electron beam, ZnMgO NPs exhibit rapid coarsening, shown in Fig. 5A. Notably, with the presence of hydrogen, ZnMgO NP thin films start observably coarsening at a lower electron dose of $2.01\times 10^{5}\;\frac{e^{-}}{{Å}^{2}}$, a dose level at which no coarsening occurred when no hydrogen were present, indicated as the yellow diamond in Fig. 4C.

It should be noted that the kinetic energy of TEM electrons is substantially higher than that of charge carriers in an operating QD-LED. Therefore, this experiment is not performed to replicate the aging conditions of a working device, but to provide a morphological investigation of the response of ZnMgO NP films. Measurements in Fig. 4 show that the electron beam irradiation leads to changes in the morphology of ZnMgO NPs, with hydrogen-species accelerating this process. To determine the atomic lattice spacing within ZnMgO NPs before and after coarsening, two-dimensional fast Fourier transforms (2D-FFT) of TEM images is carried out. As shown in Fig. 5B, atomic-resolution TEM images are taken before and after the electron beam exposure and hydrogen doping. Spatial measurement reveals a 0.28 nm d-spacing in both the pristine and the beam-exposed device, matching the expected ZnO lattice parameter (*48*–*50*). The corresponding 2D-FFT diffraction patterns exhibits a hexagonal symmetry, with four parallel vertices representing the ZnO (111) plane and two corner vertices representing the (200) plane (*51*–*53*). These observations confirm that ZnMgO NP coarsening does not alter the atomic lattice spacing.

The effect of TEM electron beam irradiation and H-doping is further tested on ZnTeSe QD-LED cross-sectional lamella. Shown in Fig. 5C, a 10-min exposure to H_2_ (3 sccm) under the TEM electron beam irradiation induces significant coarsening of ZnMgO ETL, whereas the QD EML and organic HTL remain unaffected. A pixel-by-pixel image subtraction shows significant changes only at the ETL/contact and ETL/EML interfaces. To substantiate the post-doping ZnTeSe QD layer structural consistency, we applied an identical TEM electron dose density and H_2_ concentration doping treatment to a drop-casted ZnTeSe QDs thin film on a TEM grid. Figure 5C depicts the pristine and beam/H_2_-treated blue-QDs film, respectively, where no changes are observed in the QD film. (see figs. S20 and S22 for additional proof). Furthermore, computational particle-size analysis is performed by applying a Gaussian filter that enhances edge detection to distinguish the ZnTeSe particle size, shown in fig. S23. The average QD radii in pristine (5.90 ± 0.5 nm) and doped (5.88 ± 0.5 nm) films are indistinguishable within the error. This is consistent with the overall measured mean radii (5.98 ± 0.5 nm), confirming the resilience of QD films to electron irradiation and H_2_-doping.

To test the influence of O_2_-doping (O^−^) and H_2_O-doping (OH^−^) on the morphology of the ZnMgO NPs, we tested the morphology of ZnMgO NP films before and after the TEM electron beam irradiation that followed the O_2_-doping and H_2_O-doping conditions, with data shown in Fig. 2F. After applying the same TEM electron dose amount and an identical gas doping, both O and OH species present no obvious changes to the ZnMgO NP layer, as shown in Fig. 5 (D and E).

The observation of ZnMgO NP film coarsening under electron beam irradiation in the presence of hydrogen species and not in the presence of oxygen or OH^−^, suggests that the elevated hydrogen presence, measured in the resin-free QD-LEDs, could similarly be responsible for the observed degradation of the QD-LED performance. Note that the QD films are observed to be resilient to electron iradiation under H_2_-doping, suggesting that ZnMgO NP film coarsening might dominate the measured QD-LED performance degradation.

## DISCUSSION

As progress toward the commercial viability of heavy-metal-free QD-LEDs advances, our work provides a detailed understanding of device morphological changes under operation. We identify QD-LED layer thinning and coarsening within InP and ZnTeSe Cd-free QDs EMLs, metal oxide ETL, and organic HTL. The detailed interlayer compositional analysis indicates the consequential impact of species-induced device morphology modifications. While exposure to O and OH species leads to minor changes in the ZnMgO NP film morphology, exposing ZnMgO NP films to H species and electron beam irradiation causes NPs to morphologically coarsen, similar to the ZnMgO film coarsening observed in aged QD-LEDs. To improve the device operational lifetime, we further demonstrate that acrylate-based remote resin-encapsulation treatment affects the ZnMgO NP ETL, inhibiting the morphological changes in the device layers. Presence of the acrylate-based resin suppresses the formation of hydrogen and oxygen species across the device. With resin encapsulation, over 50-fold and 5000-fold lifetime improvement in InP/ZnSe/ZnS (red) and ZnTeSe/ZnSe/ZnS (blue) QD-LEDs is observed, respectively. Although the QD chemistry can influence QD-LED operational lifetime (*16*, *40*), our present work indicates that the long-term degradation in both red and blue QD-LED architectures is also governed by the coupled chemical and morphological evolution of individual layers and neighboring interfaces. These results emphasize that stabilizing the morphology of constituent device layers and controlling the atomic species generation and migration are critical design properties for durable QD-LED operation.

Compared with conventional encapsulation strategies that primarily function as passive moisture/oxygen barriers, the demonstrated use of the remote acrylate-based resin is shown to also manage the chemical activity within the operating device layers. Although detailed degradation pathways are device-composition-dependent, the architectural insights presented in this study inform strategies to be considered in the design of both Cd-free and other-structured QD-LEDs. By visualizing how interfacial chemistry drives nanoscale device structural evolution, this work offers a broader guidance for future QD-LED designs and optimizations.

## MATERIALS AND METHODS

### Materials and QD-LED fabrications

All materials used in this study were purchased for direct use without further purification. PEDOT:PSS (AI 4083) was dissolved in aqueous solution and purchased from Ossila. Materials were kept refrigerated during storage. TFB was purchased from Montreal Optoelectronics Inc. with purity of >99%. O-Xylene (anhydrous, 97%, 1,2-dimethylbenzene) and oleic acid (technical grade, 90%) were purchased from Sigma-Aldrich. ZnMgO NPs in ethanol for usage as InP QD-LED ETLs and acrylate-based resin for encapsulation treatment were provided by Samsung Advanced Institute of Technology (SAIT). Synthesis of ZnMgO NPs solution followed the published literature (*9*). These NPs were stored in nitrogen glovebox freezer to prevent aggregation. Aluminum pellets used in the deposition of electrode materials were purchased from Kurt J. Lesker. InP/ZnSe/ZnS QD-LEDs and ZnTeSe/ZnSe/ZnS QD-LEDs for device characterizations were fabricated and encapsulated at SAIT, following the cited methods (*3*, *9*). Acrylate-based resin were provided by SAIT. Resin-treated devices were fabricated on raw QD-LEDs, where acrylate-based resin was spun cast on top of the encapsulation glass. The resin-coated encapsulation glass was then epoxy-sealed to protect the device functional area. To eliminate the effects of positive aging, all resin-encapsulated QD-LEDs were shelf-stored for over 1 week before electrical characterization to directly probe long-term negative-aging behavior. Alternatively, freshly fabricated devices were stored in an over at 60°C over 8 hours prior to current-voltage-luminance (JVL) measurements.

### QD-LED electro-optical characterizations

QD-LED current-voltage measurement was carried out using a Keithley 2636A source meter. Luminance-voltage (LV) measurement was measured using the calibrated FDS1010 Si photodiode purchased from Thorlabs Inc. The photodiode has a rise time of 65 ns, wavelength response range of 350 to 1100 nm, and 10 by 10 mm^2^ active area. Same Keithley 2636A was used as a voltage source during LV measurement.

For PL measurements, a λ = 405 nm wavelength pulsed laser (PicoQuant, LDH-P-C-405, 2 MHz repetition rate, with laser pulse duration <100 ps) or a λ = 532 nm pulsed laser (PicoQuant, P-FA-530XL, 2 MHz, with laser pulse duration <100 ps) was focused with a lens (Thorlabs, LA1978-A-ML) onto the back-focal plane of a 50× 0.7–numerical aperture air objective in an inverted microscope (Nikon, Ti-U) to generate a pseudo–wide-field excitation spot of 50 μm in diameter at the HTL/QD interface of the QD-LED. The emitted light was filtered either through a λ = 405-nm long-pass dichroic filter (Semrock, Di03-R405) or a λ = 532-nm long-pass dichroic filter (Semrock, Di02-R532) before being coupled in free-space into a spectrometer with a 150 g/mm grating (Princeton Instruments, SpectraPro-300i). For electroluminescence measurements, a constant current density of 10 mA/cm^2^ was applied to the QD-LED and no spectral filters were used. All spectra were collected with an integration time of 1000 ms.

### FIB and high-resolution transmission electron microscope

InP/ZnSe/ZnS and ZnTeSe/ZnSe/ZnS QD-LEDs cross-sectional lamellas were prepared with the Raith VELION focus ion beam scanning electron microscope (FIB-SEM) at MIT.nano. Epoxy-encapsulated cover slip of each device was removed and devices were cut into 6 mm by 6 mm pieces. To prepare the cut devices for FIB lamella milling, the devices were first coated with 5 nm of carbon and a layer of Pt. FIB process then followed the sequence of trenching, undercutting, nanomanipulator inserting, Pt connection, lifting up, TEM grid positioning, lamella attaching, Pt coating, lamella milling, lamella thinning, and polishing (again see fig. S5). Lamellas were etched using a gold ion beam at 35 kV for milling and 5 kV for polishing.

The milled LED lamella was then transferred into the Thermo Fisher Scientific Themis Z G3 aberration-corrected scanning transmission electron microscope at MIT.nano for imaging. The images were captured under a current of 100 pA with a probe size of 0.7 Å.

### Time-of-flight secondary ion mass spectrometry

Positive high mass resolution depth profiles were performed using a TOF-SIMS NCS instrument, which combines a TOF.SIMS5 instrument (ION-TOF GmbH, Münster, Germany) and an in situ scanning probe microscope (NanoScan, Switzerland) at Shared Equipment Authority from Rice University. The analysis field of view was 100 by 100 μm^2^ (Bi_3_^+^ @ 30 keV, 0.3 pA) with a raster of 128 by 128 along the depth profile. A charge compensation with an electron flood gun has been applied during the analysis. An adjustment of the charge effects has been operated using a surface potential. The cycle times were fixed to 100 μs (corresponding to *m*/*z* = 0 to 911 a.m.u mass range). The sputtering raster was 450 by 450 μm^2^ (Cs^+^ @ 1 keV, 85 nA). The beams were operated in noninterlaced mode, alternating one analysis cycle and three frames of sputtering per cycle followed by a pause of 3 s for the charge compensation. The depth calibrations have been established using the interface tool in SurfaceLab version 7.5 software from ION-TOF GmbH to identify the different interfaces and based on the measured thicknesses before the analysis.

To better process the data, the collected data are automatically normalized against the Cs^+^ primary ion beam intensity. The collected spectra in Fig. 2F are then calibrated with the O level in the ITO layer as reference.

### Device operational self-heating simulation

The thermal simulation of the operational QD-LED is carried out through MATLAB. To get a conservative estimate of overall heating, the device was approximated as a single glass with the same dimensions as the encapsulated device (35 by 35 by 1.6 mm). We assume that 100% of the device power is converted to thermal power. The thermal heating is modeled as a 1 mm by 2 mm by 10 μm (blue QD-LED) and 2 mm by 2 mm by 10 μm (red QD-LED) uniform heat source in the center of the glass slab. In addition, the device is assumed to be floating in air during operations.

### In situ transmission electron microscopy with hydrogen doping

#### Sample preparation

In preparation for in situ TEM imaging, the NPs were removed from the freezer and drop-casted on a Ted Pella copper TEM grid with ultrathin carbon film on lacey carbon. The grids were baked in a vacuum oven at 50°C for 10 hours to evaporate the ethanol solvent and to prevent contamination during TEM imaging.

#### In situ transmission electron microscope

Samples were exposed to hydrogen gas and imaged on a Hitachi HF500 environmental TEM. Without hydrogen gas in the system, the vacuum level of the imaging chamber was 1.7 × 10^−5^ Pa. Hydrogen gas was flowed into the imaging chamber at 3 sccm. With hydrogen in the chamber, the chamber pressure increased to around 10^−2^ Pa. The ultra-high resolution (UHR) mode has a beam current of 15 pA. The HR mapping mode has a beam current of 100 pA. Microscope probe size is 0.8 Å in diameter.

### Total electron dose calculation and relevance device current

Total electron dose was calculated following the equation: $R[\frac{e^{-}}{{Å}^{2}s}]=\frac{J\left[\frac{C}{s}\right]}{e^{-}\left[\frac{C}{e^{-}}\right]\times A\left[{Å}^{2}\right]}$ where *R* represents the electron beam dose rate, *J* represents the microscope current density, in the unit of pA or column per second, and *A* represents the total probe size. Probe size was calculated as a square shape. The total electron dose per image $\left[\frac{e^{-}}{{Å}^{2}}\right]=R\times \frac{t}{\text{per}{\text{pixel}}^{2}}$. Here, *t* is the total time where electron beam passing through per area pixel. Same equation was applied when linking electron dose causing ZnMgO NPs to coarsen to the total current applied to QD-LEDs during operation. The total amount of charge that flowed through an aged QD-LED follows: $Q\left[\frac{e^{-}}{{Å}^{2}}\right]=\frac{I\left[\frac{C}{s}\right]}{1e^{-}\left[\frac{C}{e^{-}}\right]\times A\left[{Å}^{2}\right]}\times t_{\text{driven}}\left[s\right]$, where *I* is the constant current applied to the device, *A* is the QD-LED operation area, and $t_{\text{driven}}$ is the total amount of operational aging time.

### Lambertian EQE calculation

The Lambertian EQE calculation follows the cited method (*35*): $\text{EQE}=\frac{P_{\text{out}}/\left(\frac{\mathrm{hc}}{\lambda_{\text{peak}}}\right)}{J_{\text{LED}}/q}$ where $P_{\text{out}}\left[W\right]=\frac{L\left[A\right]}{f\times R\left[\frac{A}{W}\right]}$ and f is the directionality correlation factor. From the J-V-L setup, the distance between the photo diode and the LED is $d_{\text{PD}}=50\text{mm}$. The directionality correlation factor is calculated through $f=\frac{P_{\text{PD}}}{P_{\text{out}}}=\frac{\int_{0}^{\theta_{\text{PD}}}I\left(\theta \right)d\theta }{\int_{0}^{\frac{\pi }{2}}I\left(\theta \right)d\theta }={\text{sin}}^{2}\left(\text{arctan}\left(\frac{r_{\text{PD}}}{d_{\text{PD}}}\right)\right)$. The light power collected by the photodiode is obtained through *R*, responsivity, $P_{\text{PD}}\left[W\right]=\frac{L\left[A\right]}{R\left[\frac{A}{W}\right]}$ at 630 nm for red and at 475 nm for blue. The LED output power is calculated through $P_{\text{out}}\left[W\right]=\frac{P_{\text{PD}}\left[W\right]}{f}$, and the final EQE follows the equation: $\text{EQE}=\frac{P_{\text{out}}/E_{\text{Avg}}}{J_{\text{LED}}/q}$ where $E_{\text{Avg}}=\frac{\mathrm{hc}}{\lambda_{\text{peak}}}$.

## Supplementary Materials

### The PDF file includes:

Figs. S1 to S23 Demo Codes Legends for movies S1 and S2
