# Supplementary material for: Morphological and chemical changes in Cd-free colloidal QD-LEDs during operation
Source: Sci Adv. 2026 Jul 10;12(28):eaec8208. doi: 10.1126/sciadv.aec8208 (PMC13353410; doi:10.1126/sciadv.aec8208)
Supplement: Supplementary file 1 — Figs. S1 to S23 Demo Codes Legends for movies S1 and S2 [file sciadv.aec8208_sm.pdf]

Science Advances
AAAS

Supplementary Materials for

Morphological and chemical changes in Cd-free colloidal QD-LEDs during operation

Ruiqi Zhang *et al.*

Corresponding author: Vladimir Bulović, bulovic@mit.edu

Sci. Adv. **12**, eaec8208 (2026)
DOI: 10.1126/sciadv.aec8208

The PDF file includes:

Figs. S1 to S23
Demo Codes
Legends for movies S1 and S2

Other Supplementary Material for this manuscript includes the following:

Movies S1 and S2

Supplementary Text and Figures

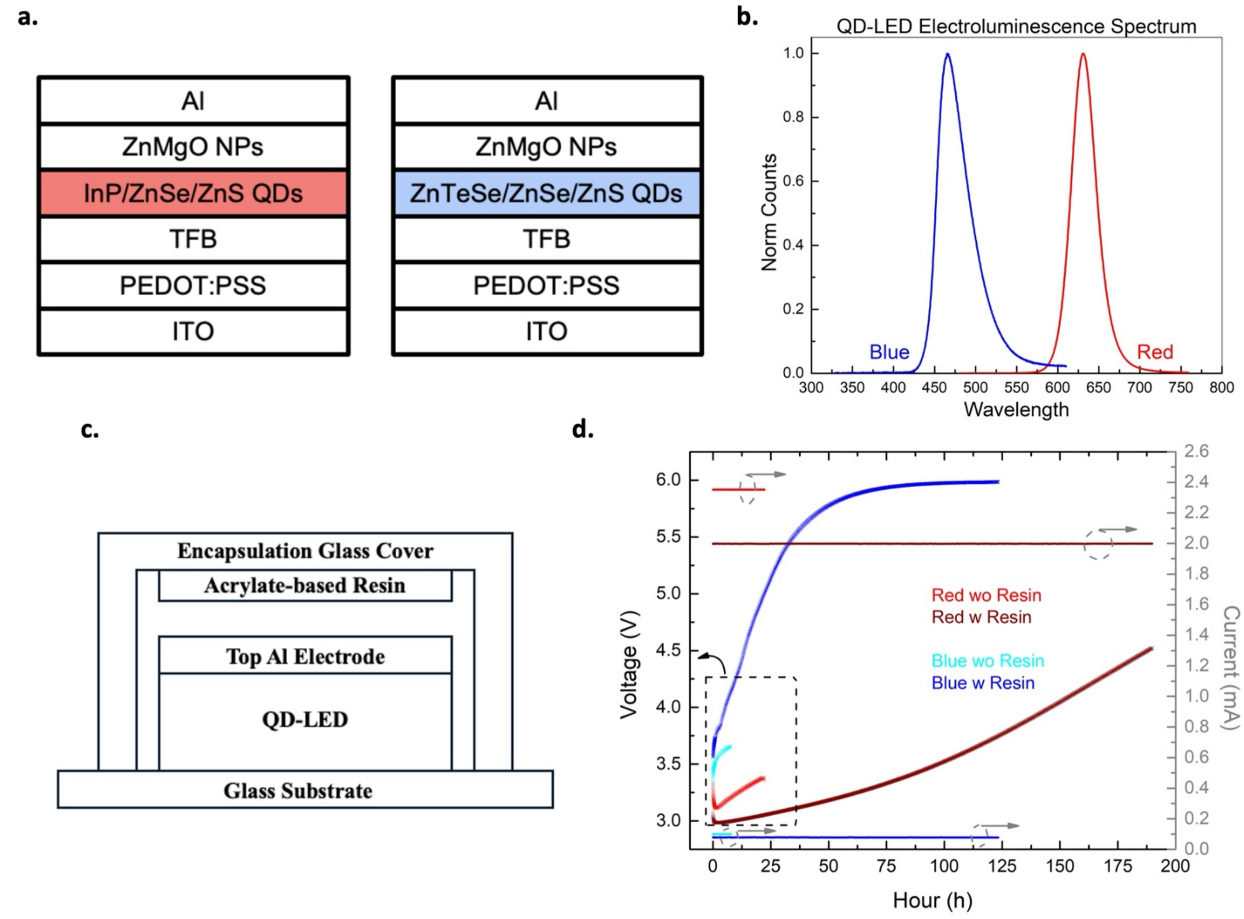

Fig. S1. Device Structure and Operation Spectra

a. Red (left) and blue (right) QD-LED structure. **b.** Normalized electroluminescence (EL) spectrum peaks. **c.** Side-view of the resin-encapsulated device structure. **d.** Voltage behavior under constant current input during device operation.

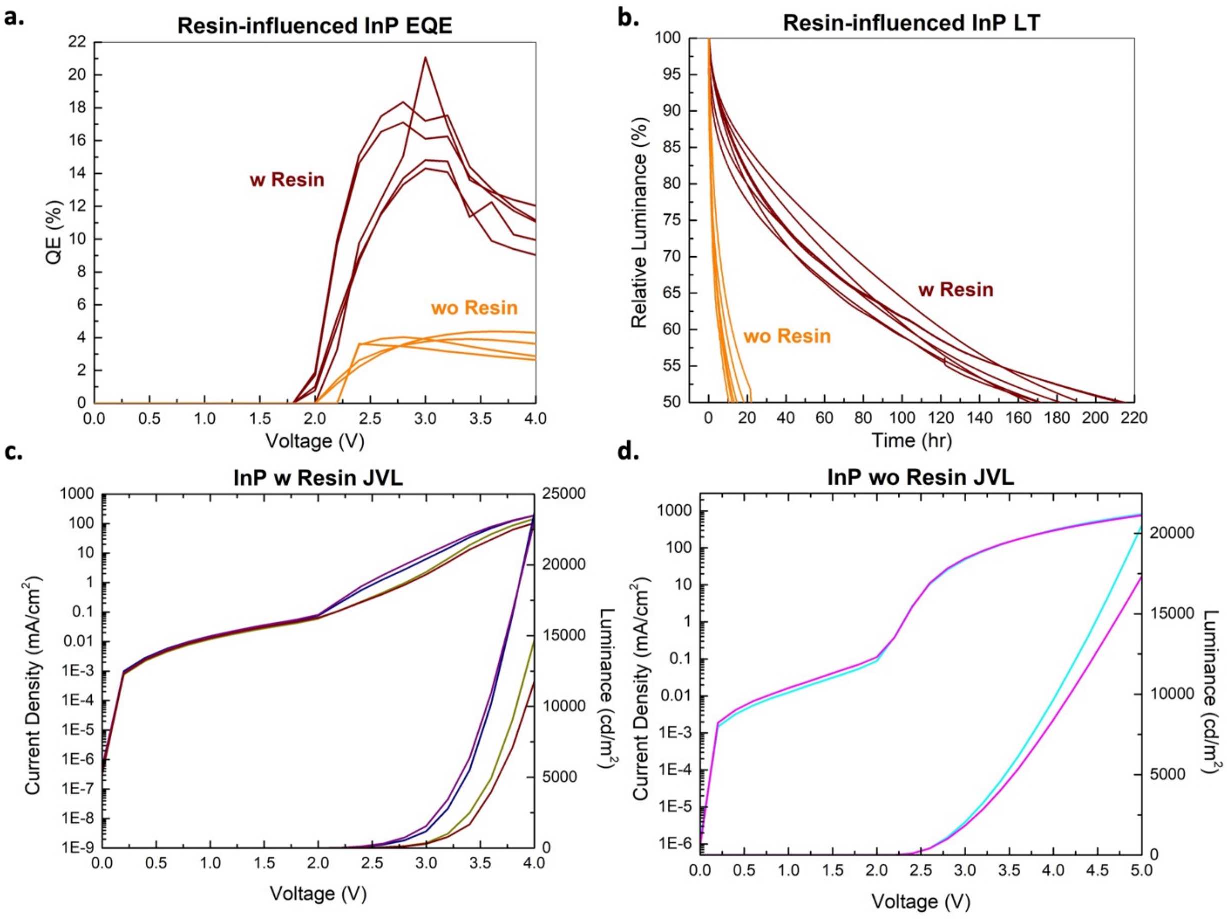

Fig. S2. InP/ZnSe/ZnS Device Performance

a. Resin-induced influence on device EQE. **b.** Resin-induced influence on device T50 lifetime. **c.** Resin-encapsulated InP devices JVL. **d.** Resin-free encapsulated InP devices JVL.

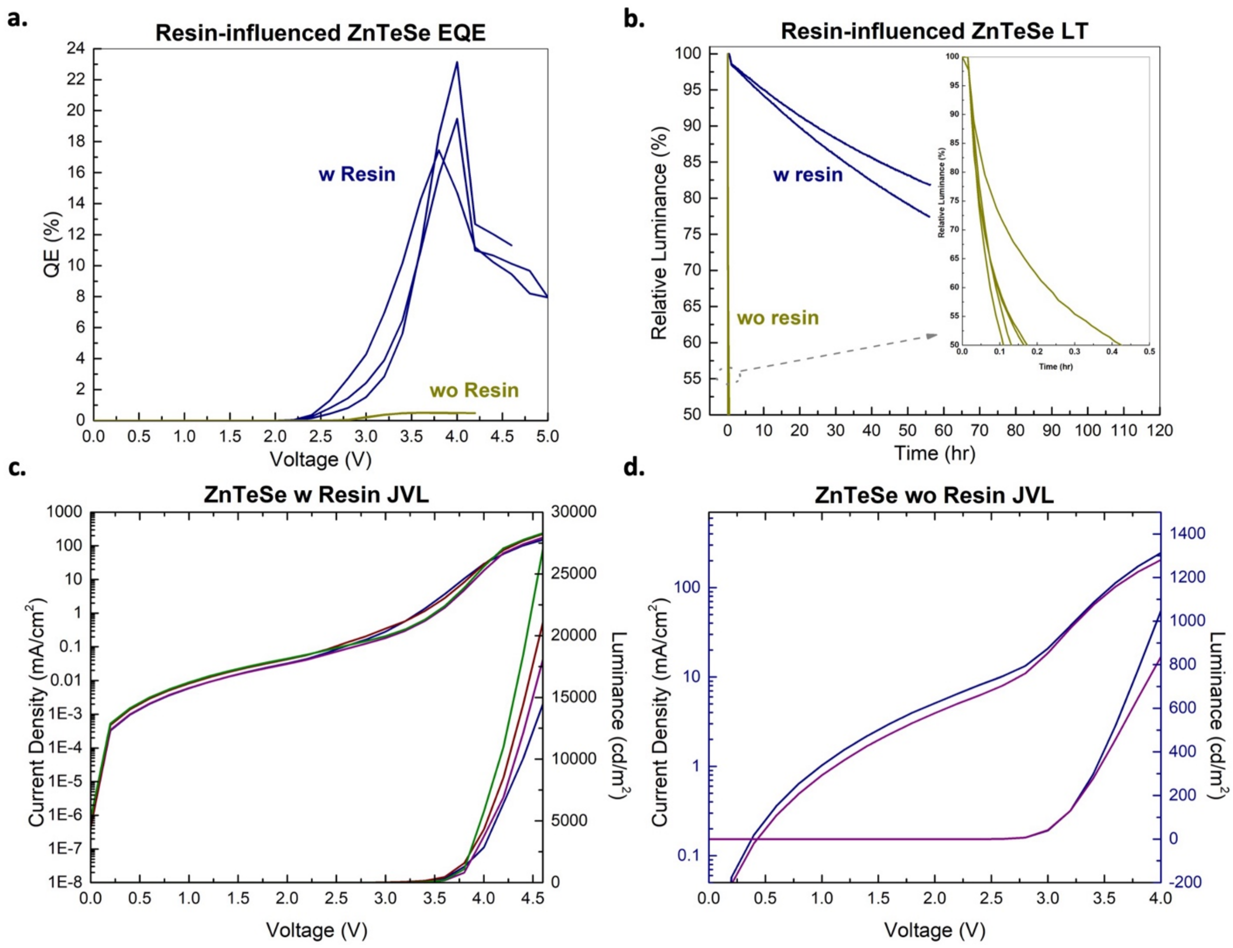

Fig. S3. ZnTeSe/ZnSe/ZnS Device Performance

a. Resin-induced influence on device EQE. **b.** Resin-induced influence on device T50 lifetime. **c.** Resin-encapsulated ZnTeSe devices JVL. **d.** Resin-free encapsulated ZnTeSe devices JVL.

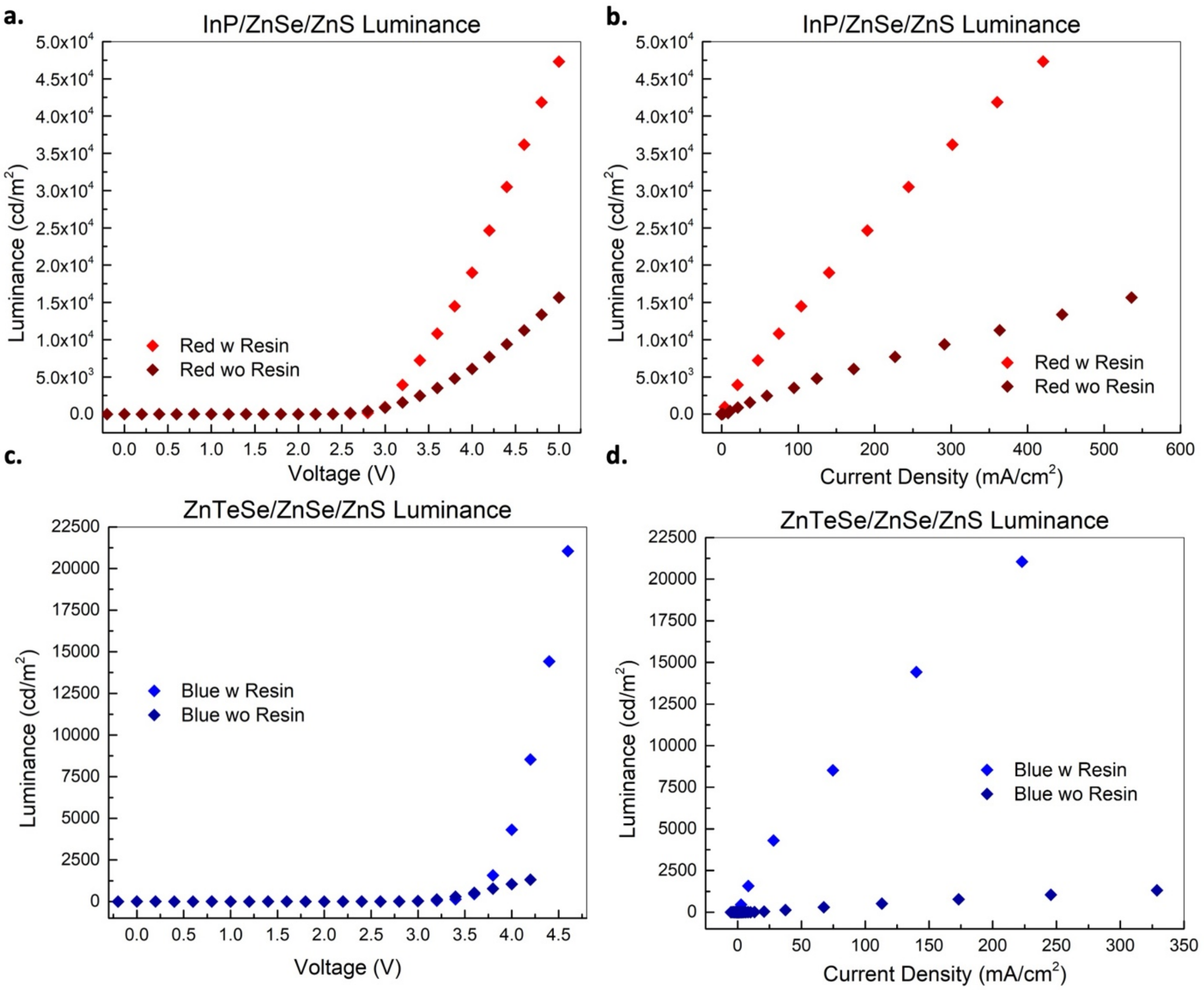

Fig. S4. Luminance Profile of InP-based and ZnTeSe-based QD-LEDs

Response of InP-based red QD-LEDs **a.** Luminance vs. voltage. **b.** Luminance vs. current density. Response of ZnTeSe-based blue QD-LEDs **c.** Luminance vs. voltage. **d.** Luminance vs. current density.

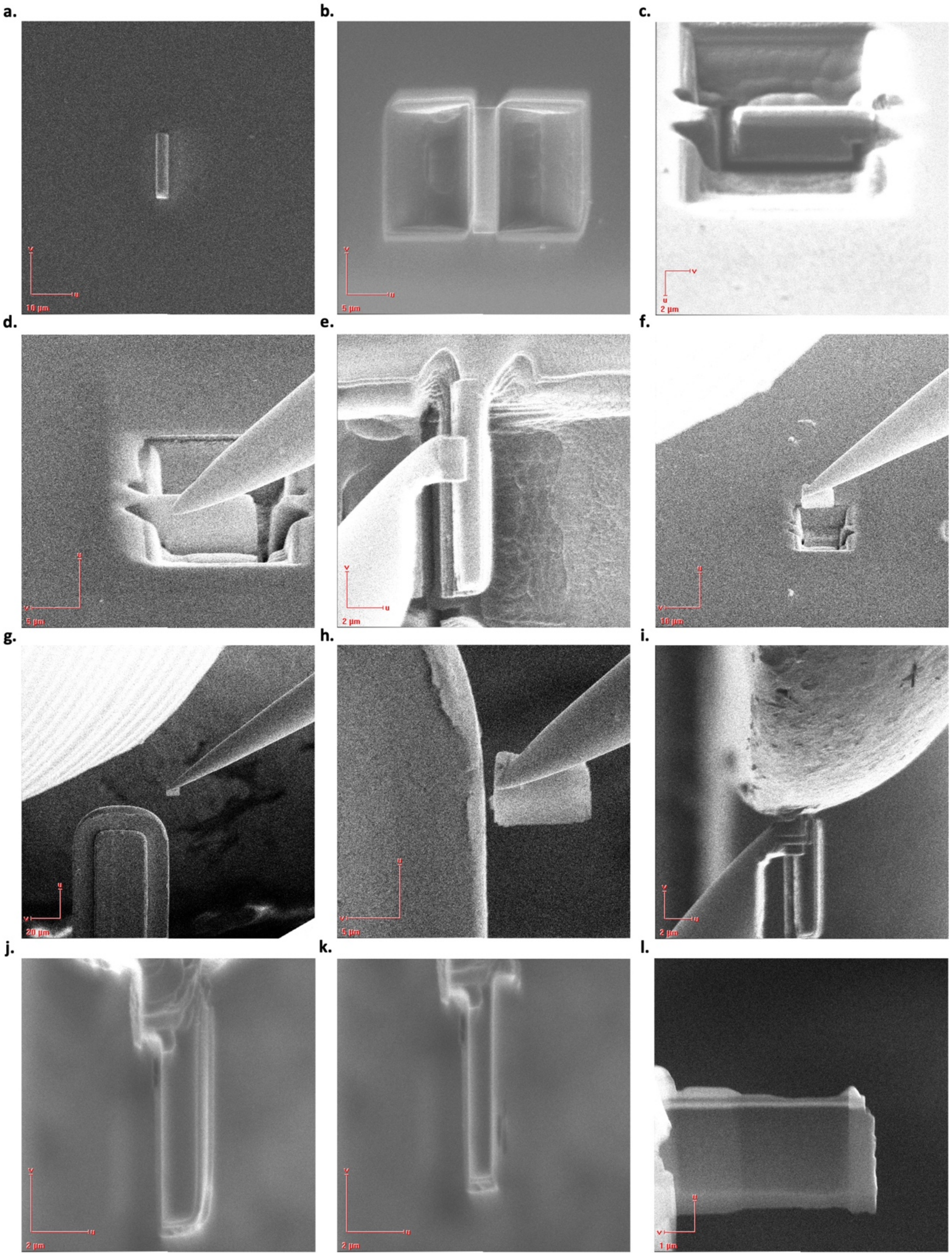

Fig. S5. LED Lamella Preparation with Focused Ion Beam (FIB) Cross-sectioning

QD-LED cross-section lamella preparation steps with FIB. **a.** Pt coating. **b.** trenching. **c.** under-cutting. **d.** nano-manipulator inserting. **e.** Pt connection. **f.** lifting-up. **g.** driving to TEM grid. **h.** attaching. **i.** Pt coating. **j.** lamella milling. **k.** thinning and polishing. **l.** final lamella prepared.

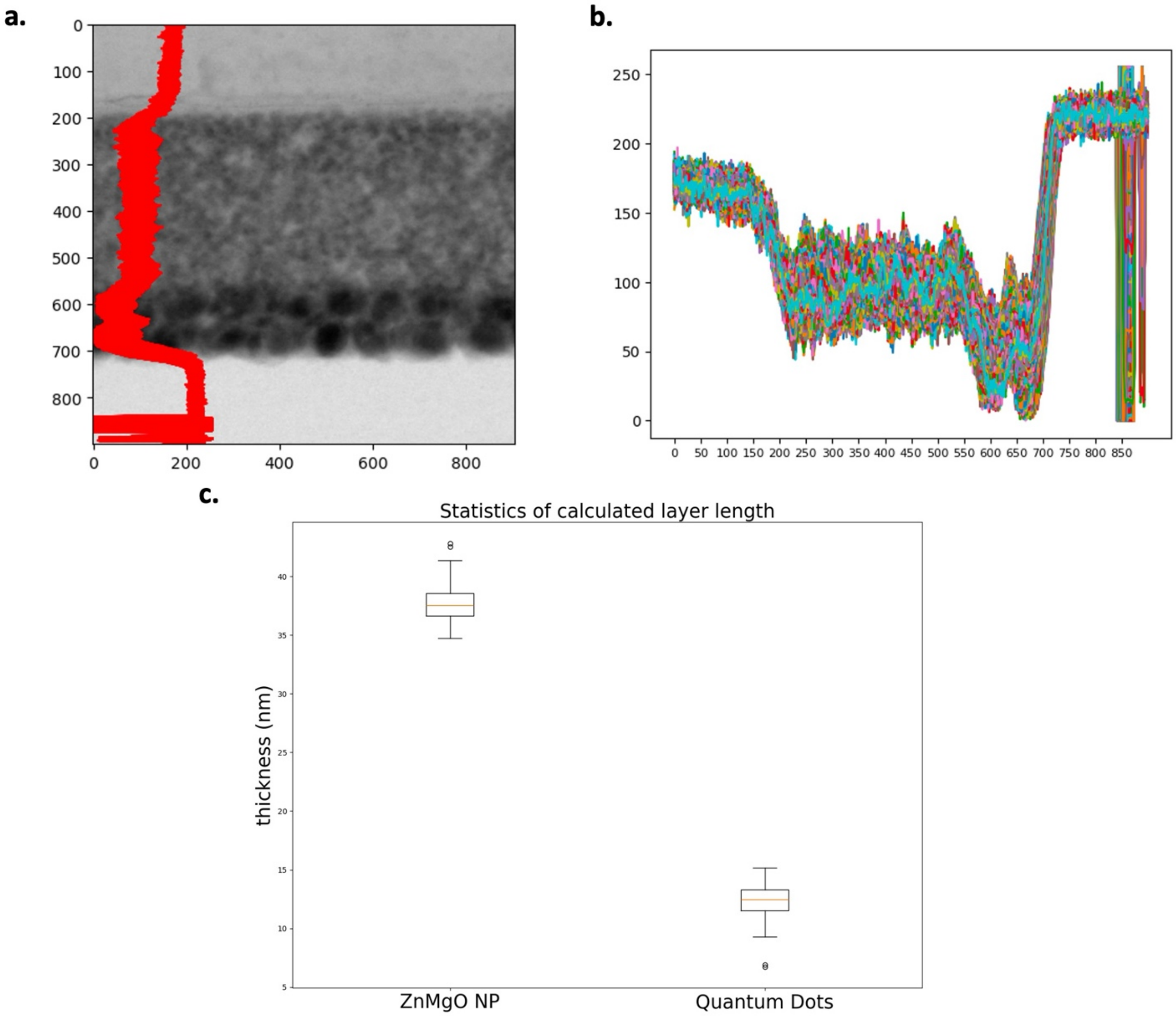

Fig. S6. QD-LED Layer Thickness Determination from TEM Images

a. Grayscale TEM image obtained from Fig. 2b InP pristine image (900 wide by 906 high). The set of red-color lines plotted over the left-side of the image are described in **b**. **b.** Brightness of pixels in the grayscale image ranges from 0 to 255. Plot shows the brightness for each vertical line of pixels of the image in figure (a). **c.** For each line of pixels plotted in **b**, we can determine the midpoint along the falling and rising brightness edges, which we assign to the edges of the QD and ZnMgO layers. We use the distances between the midpoints as the measurement of the thicknesses of QD and ZnMgO layers. The yellow line represents the average extracted thickness, with error bars presented in black.

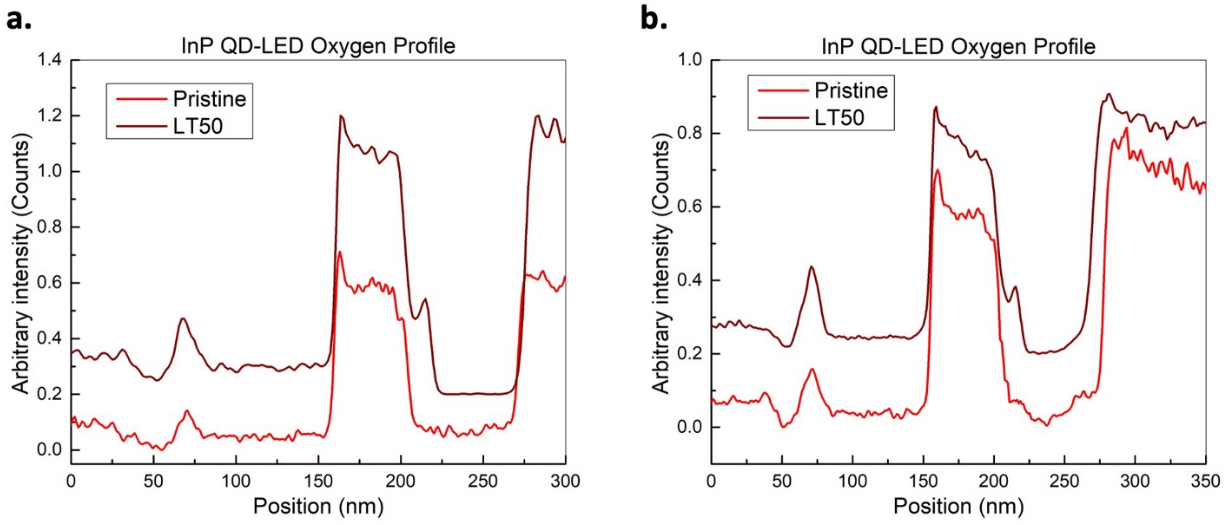

Fig. S7. Reproducibility of InP/ZnSe/ZnS QD-LED EDS Oxygen Profile in QD-LEDs without resin packaging

a. Energy dispersive X-ray Spectroscopy (EDS) Oxygen spectrum of another fabricated red InP/ZnSe/ZnS QD-LED without resin packaging. **b.** Oxygen profile of InP/ZnSe/ZnS QD-LED as presented in Main Fig. 2d, showing a similar oxygen distribution after device aging.

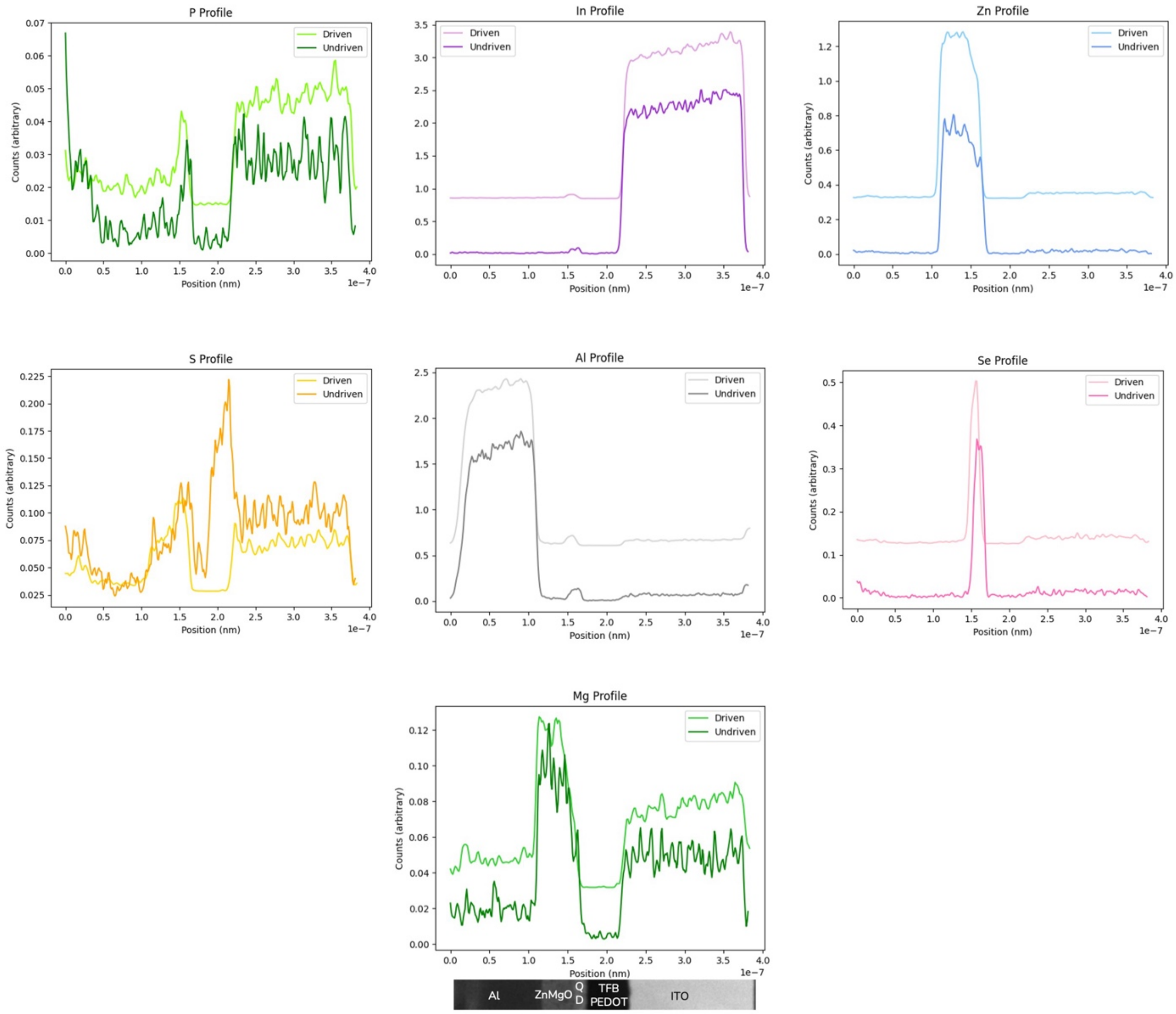

Fig. S8. Resin-free InP/ZnSe/ZnS QD-LED EDS Profiles

Energy dispersive X-ray Spectroscopy (EDS) spectra of P, In, Zn, S, Al, Se and Mg as a function of position within the InP/ZnSe/ZnS QD-LED layers.

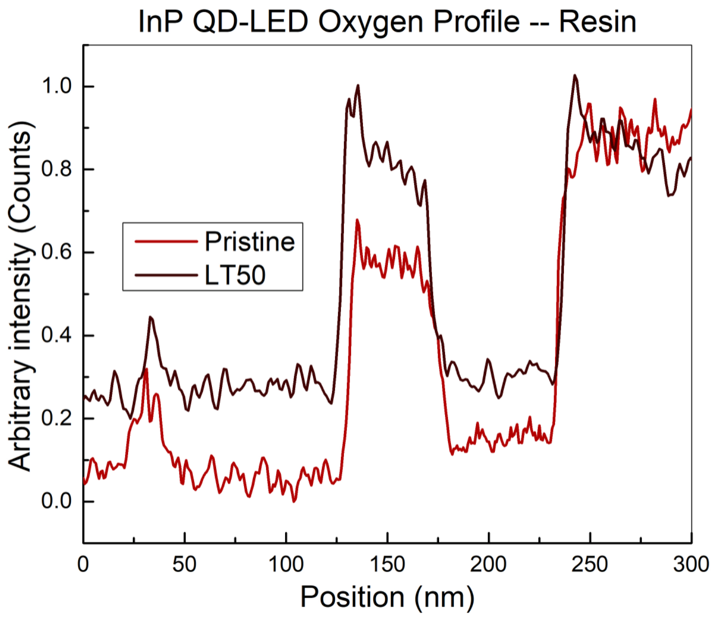

Fig. S9. Resin-encapsulated InP/ZnSe/ZnS QD-LED EDS Oxygen Profile

Energy dispersive X-ray Spectroscopy (EDS) oxygen spectrum of a red InP/ZnSe/ZnS QD-LED packaged with acrylate-based resin. No extra oxygen peak is present in the QD layer after aging.

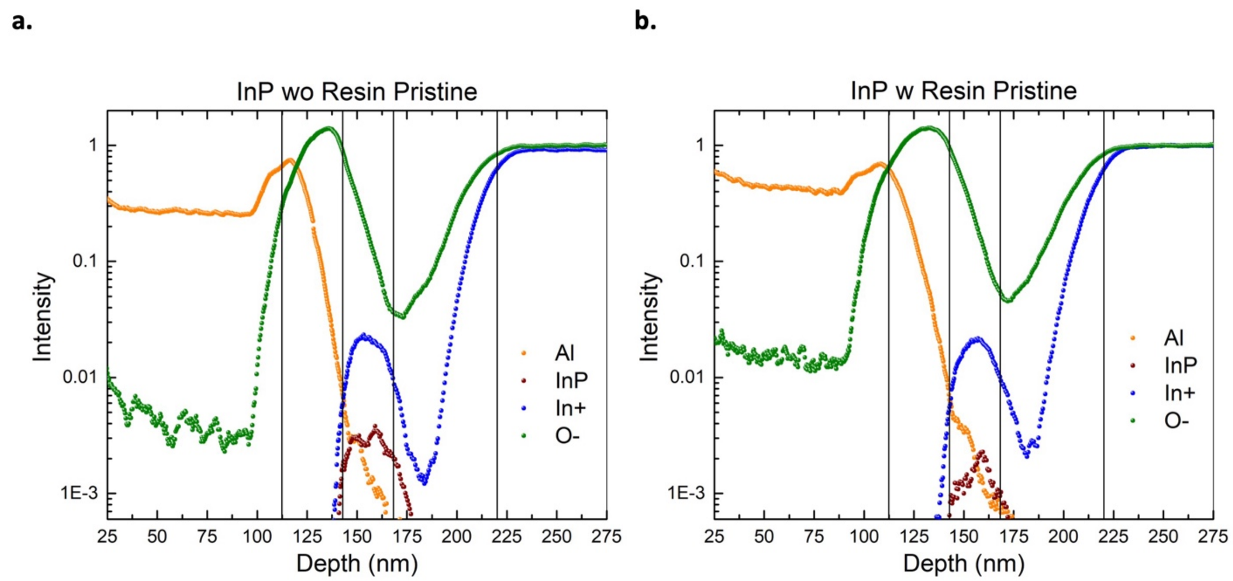

Fig. S10. TOF-SIMS Profile Indicating InP/ZnSe/ZnS QD-LEDs Layers

TOF-SIMS spectrum of characteristic elements in InP QD-LED layers for **a.** pristine device without resin-encapsulation and **b.** pristine device with resin-encapsulation.

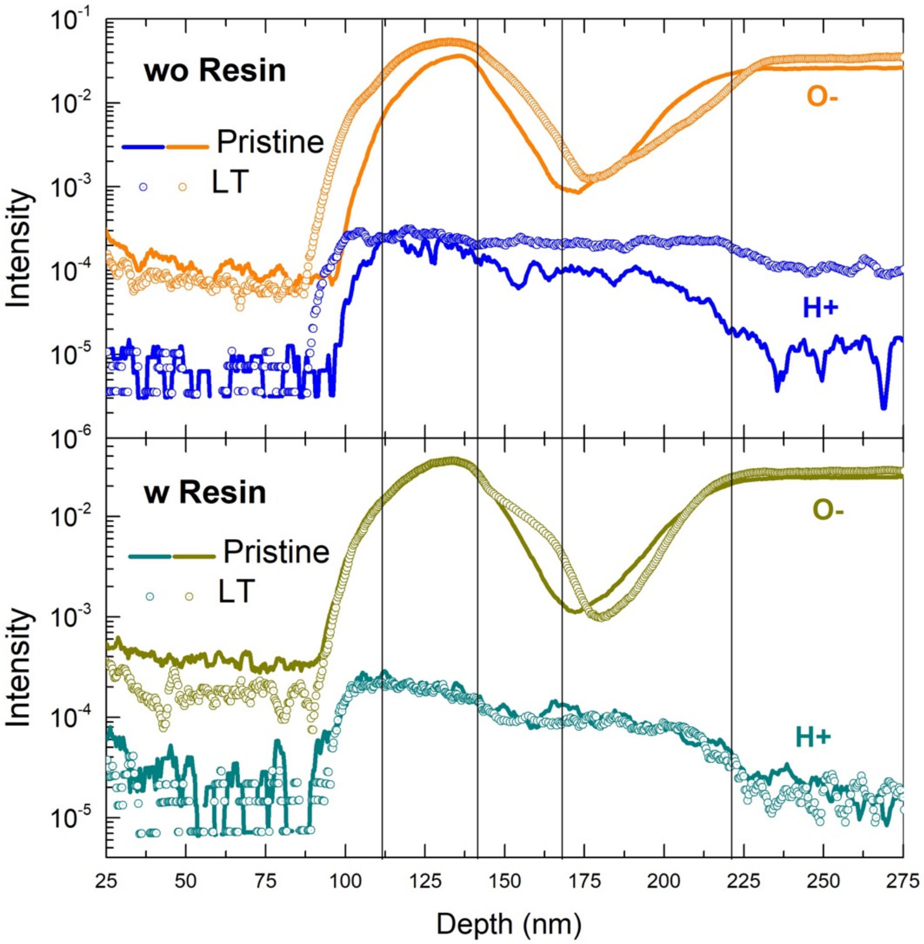

Fig. S11. Raw InP/ZnSe/ZnS QD-LED TOF-SIMS Profile

Raw TOF-SIMS spectrum of H^+ and O^- before normalization process as indicated in Figure 2f.

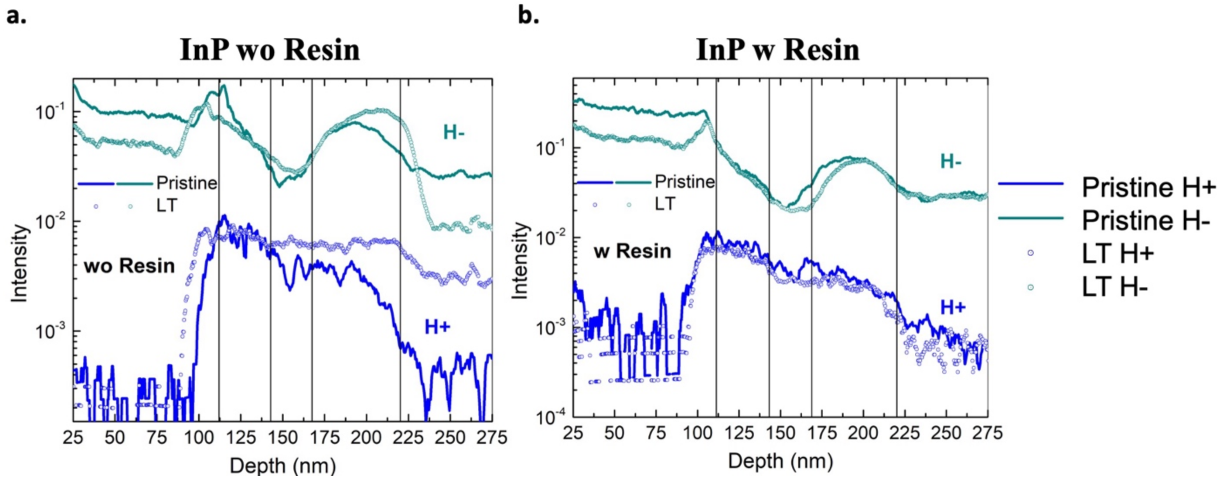

Fig. S12. InP/ZnSe/ZnS LED w/ & w/o resin TOF-SIMS spectrum of Hydrogen Species

TOF-SIMS spectrum of hydrogen (H^+ and H^-) on **a.** resin-free and **b.** resin-encapsulated InP/ZnSe/ZnS QD-LEDs. Blue color indicates TOF-SIMS distribution of H^+. Cyan color indicates TOF-SIMS distribution of H^-. Solid lines represent spectra from pristine devices, while scatter plots represent LT-aged devices. A clear inhibition of hydrogen generation is observed in resin-encapsulated devices.

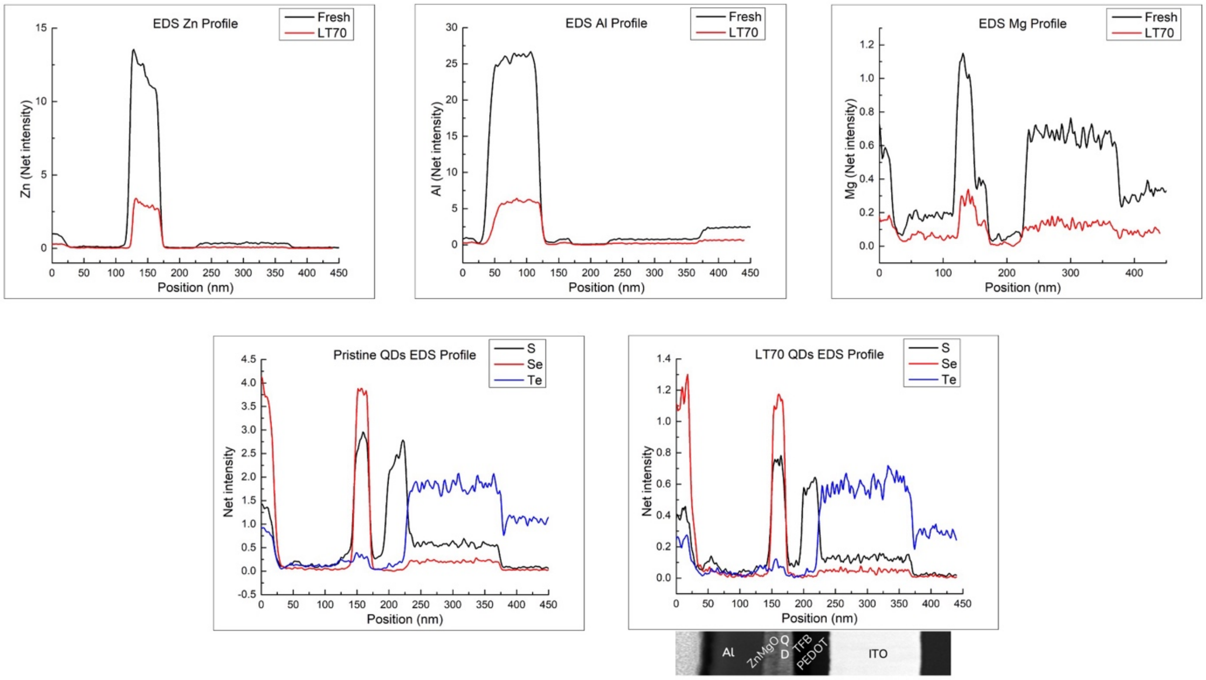

Fig. S13. Resin-free ZnTeSe/ZnSe/ZnS QD-LED EDS Profiles

Energy dispersive X-ray Spectroscopy (EDS) spectra of Zn, Al, Mg, S, Se and Te as a function of the ZnTeSe/ZnSe/ZnS QD-LEDs layers.

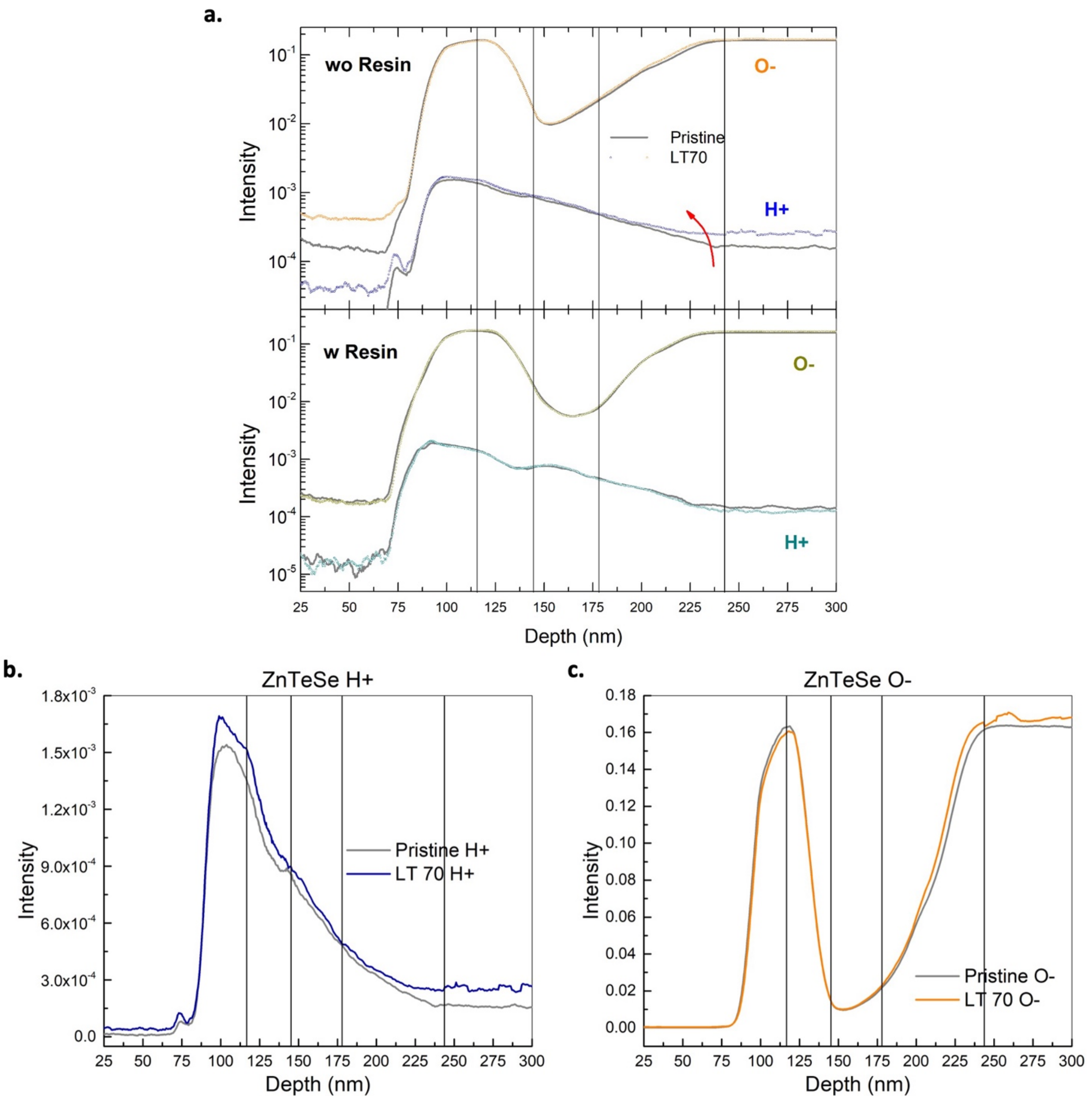

Fig. S14. ZnTeSe/ZnSe/ZnS QD-LED w/ & w/o resin TOF-SIMS spectrum of Hydrogen and Oxygen Species

a. ToF-SIMS spectra of resin-free and resin-encapsulated ZnTeSe/ZnSe/ZnS QD-LEDs. **b.** and **c.** Replotted Figure **a.** for H species and O species in linear scale.

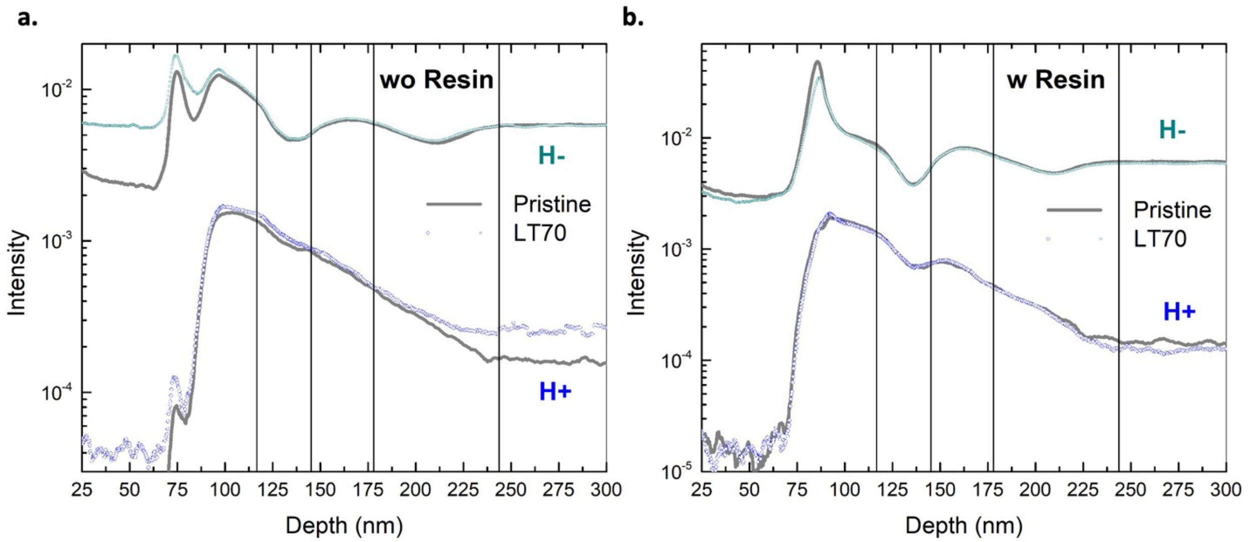

Fig. S15. ZnTeSe/ZnSe/ZnS LED w/ & w/o resin TOF-SIMS spectrum of Hydrogen Species

TOF-SIMS spectrum of hydrogen (H^+ and H^-) on **a.** resin-free and **b.** resin-encapsulated ZnTeSe/ZnSe/ZnS QD-LEDs. Blue color indicates TOF-SIMS distribution of H^+ after LT70 aging. Cyan color indicates TOF-SIMS distribution of H^- after aging. Solid gray lines represent spectra from pristine devices, while scatter plots represent LT-aged devices.

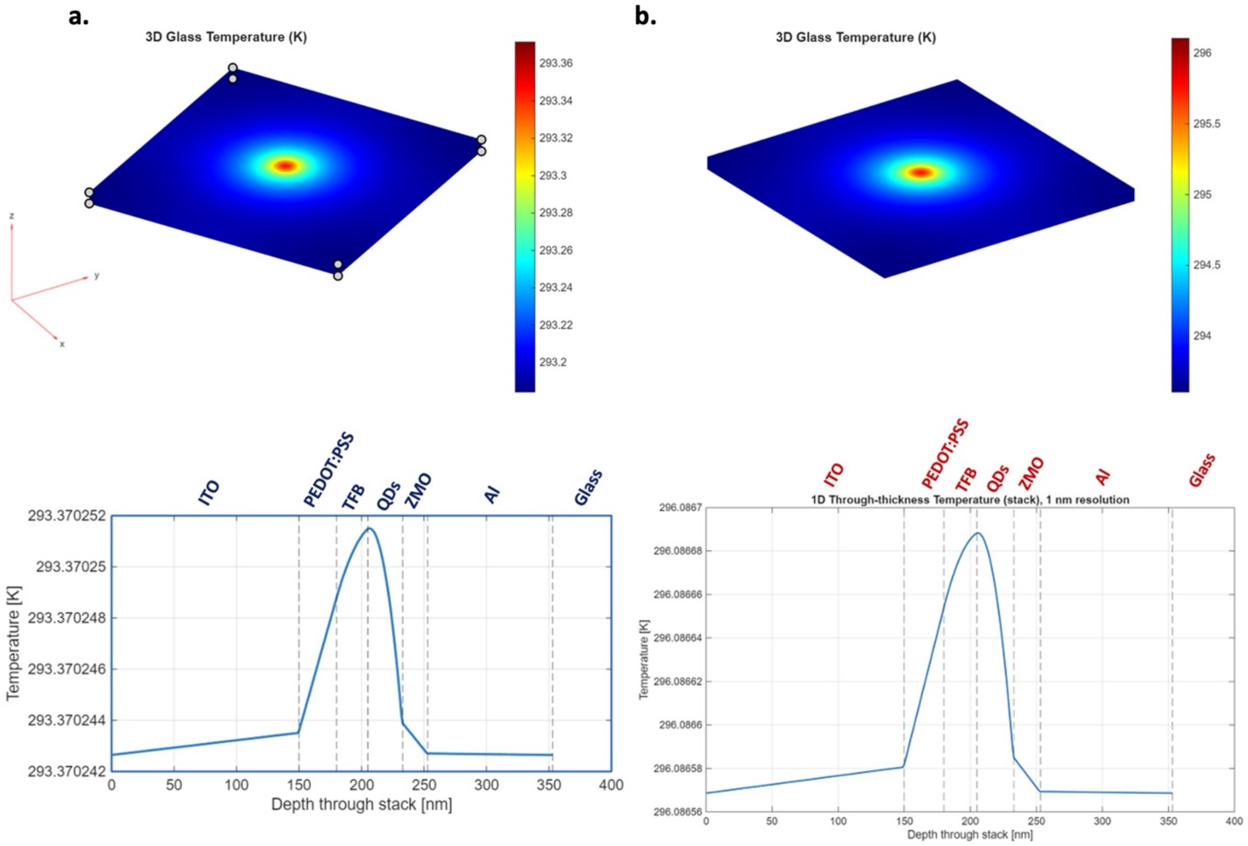

Fig. S16. Simulation Profile of QD-LED Self-heating under Degradation Operation Steady-state

a. Lateral thermal simulation result of a 1-by-2 mm^2 ZnTeSe-based QD-LED on a bare glass substrate (upper). Lateral heat distribution across all layers in an operational ZnTeSe-based QD-LED (lower). **b.** Lateral thermal simulation result of a 2-by-2 mm^2 InP-based QD-LED on a bare glass substrate (upper). Lateral heat distribution across all layers in an operational InP-based QD-LED (lower).

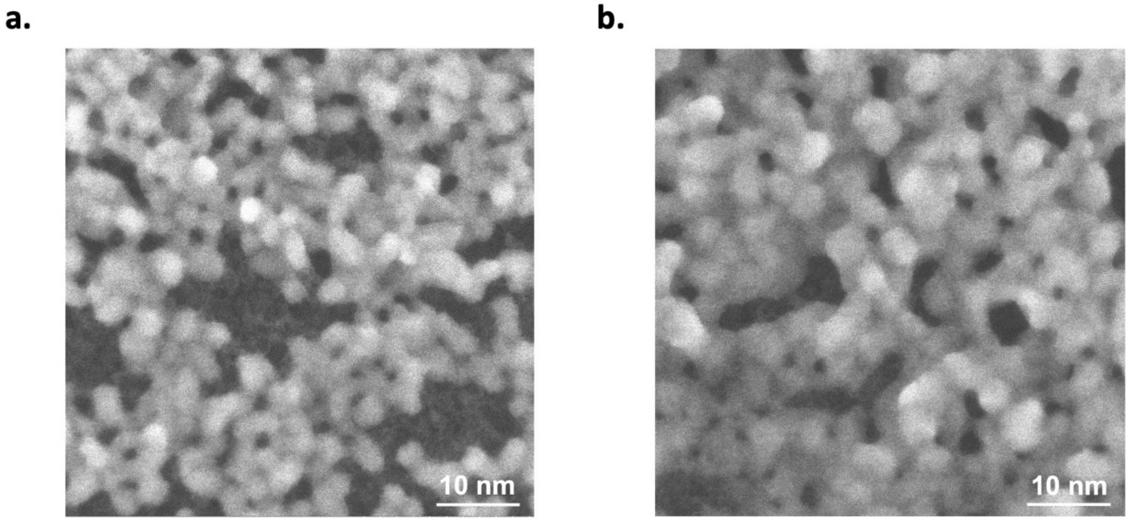

Fig. S17. Beam-irradiation-induced ZnMgO NPs coarsening

Beam irradiation on ZnMgO NPs. **a.** pristine, and **b.** irradiated ($2.24\times 10^6\ \frac{e^-}{Å^2}$) ZnMgO NPs. Both images are acquired in the same region.

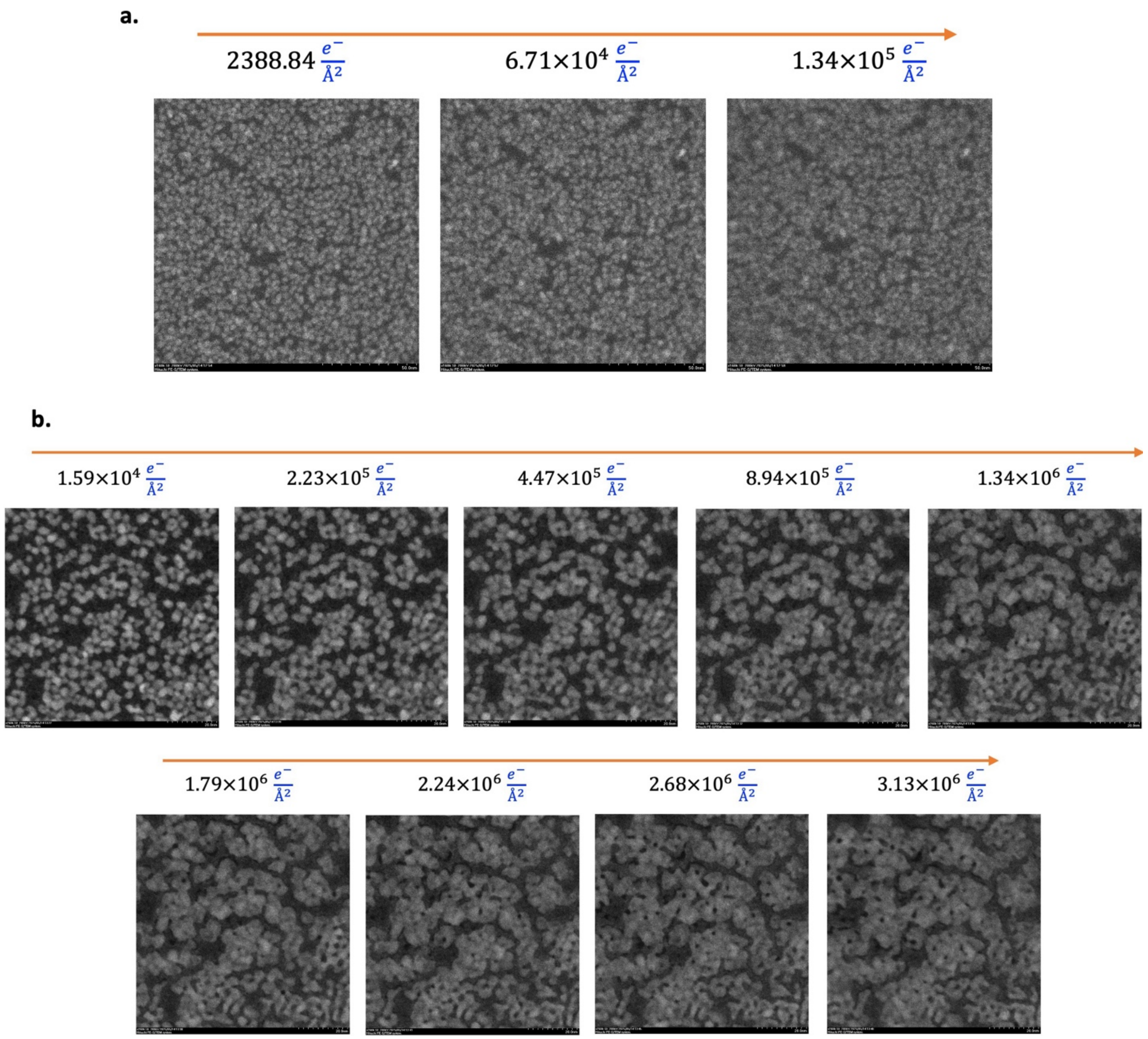

Fig. S18. Impact of the TEM Electron Beam Dose-Density on ZnMgO NPs Coarsening

Evolution of dose-dependent ZnMgO NP coarsening **a.** Region 1, beam-induced particles evolution from 2389 $\frac{e^-}{Å^2}$ to 1.34×10^5 $\frac{e^-}{Å^2}$, no coarsening observed. **b.** Region 2, beam-induced particles evolution from 1.59×10^5 $\frac{e^-}{Å^2}$ to 3.13×10^6 $\frac{e^-}{Å^2}$.

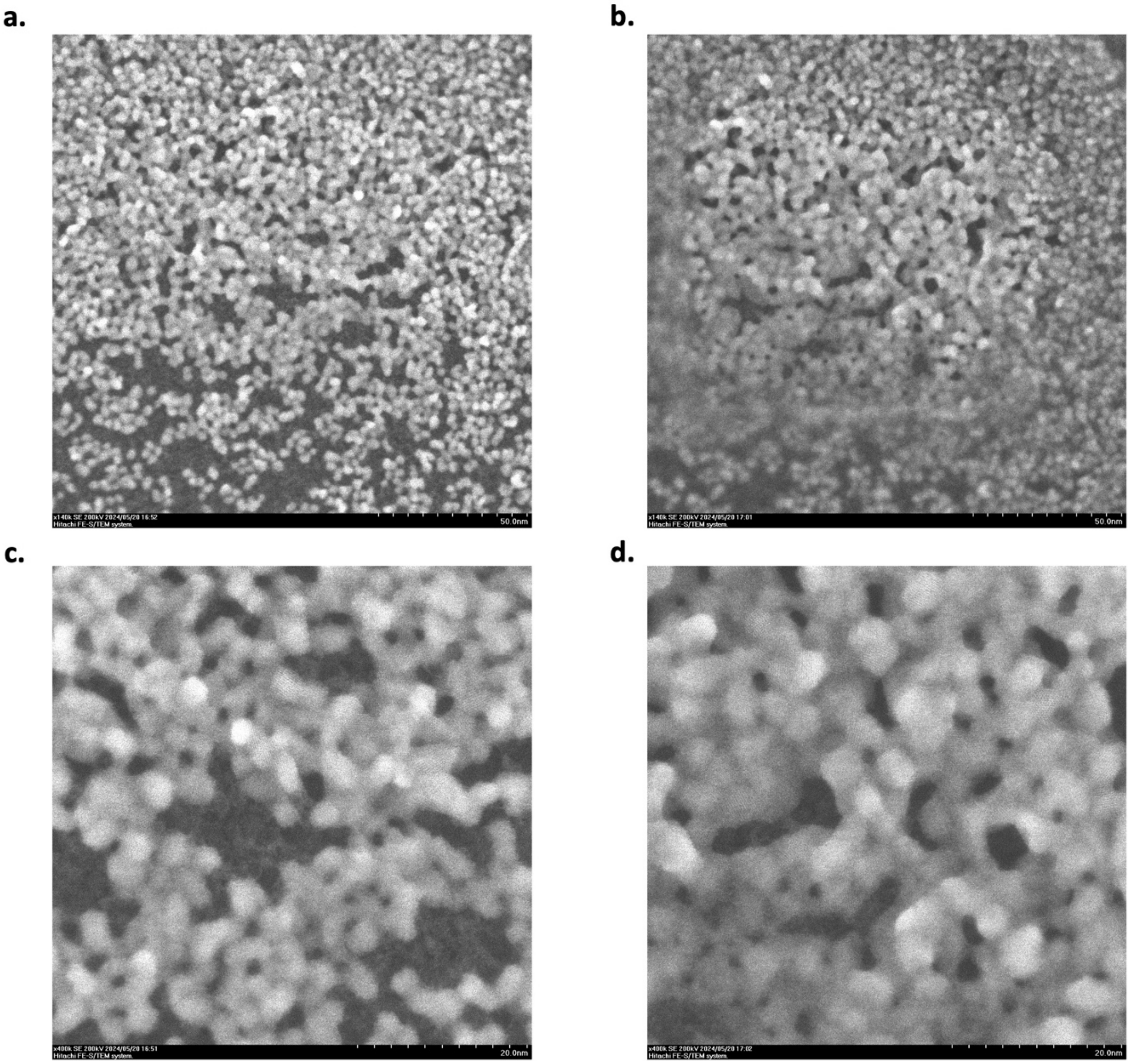

Fig. S19. Influence of Electron Beam Irradiation on ZnMgO Nanoparticle Thin Films

Another example of influence of beam irradiation on ZnMgO NPs thin film. **a.** HR-TEM image of pristine drop-casted ZnMgO NPs thin film. **b.** HR-TEM image of same region beam-irradiated ZnMgO NPs thin film. **c.** Zoomed in high resolution HR-TEM image acquired in **a** (same as Fig. S17 a). **d.** Zoomed in high resolution HR-TEM image acquired in **b** (same as Fig. S17 b).

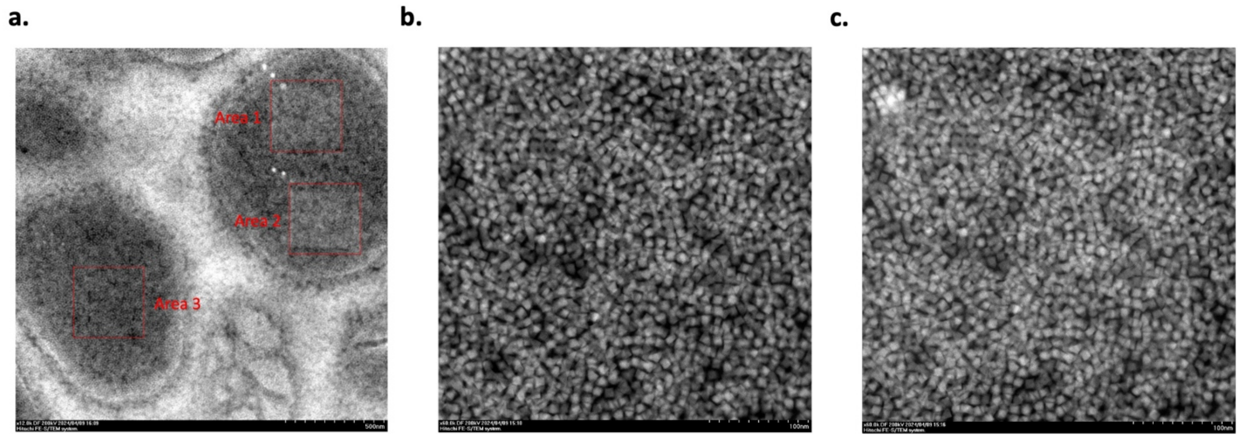

Fig. S20. Influence of Electron Beam Irradiation on ZnTeSe/ZnSe/ZnS QDs

a. HR-TEM images of drop-casted ZnTeSe/ZnSe/ZnS QD thin films. Area 1 is beam-irradiated only (with detailed results shown in parts **b-c** of this figure). Area 2 is doped with H_2 while electron beam irradiated (with detailed results shown in Fig.S21). Area 3 is H_2-doped only (with detailed results shown in Fig.S22). **b.** Pristine blue QD-LED thin film in Area 1. **c.** Beam irradiated (with electron beam dose of $3.13 \times 10^6\ \frac{e^-}{\text{Å}^2}$) ZnMgO NPs thin film in the same region of Area 1. QD film coarsening is not observed.

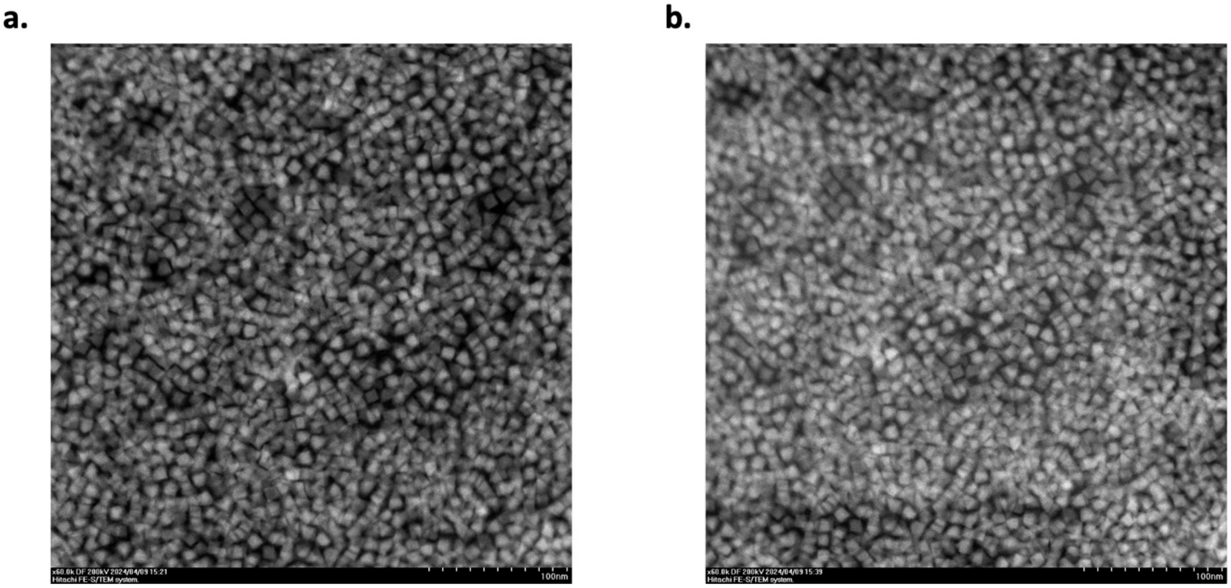

Fig. S21. Influence of H_2-doping with Electron Beam Irradiation on ZnTeSe/ZnSe/ZnS QDs

a. Pristine blue ZnTeSe/ZnSe/ZnS QD thin film in Area 2. **b.** Area 2 doped with H_2 while electron beam irradiated (with electron beam dose of $3.13 \times 10^6\ \frac{e^-}{\text{Å}^2}$) in the same region as **a.** QD film coarsening is not observed.

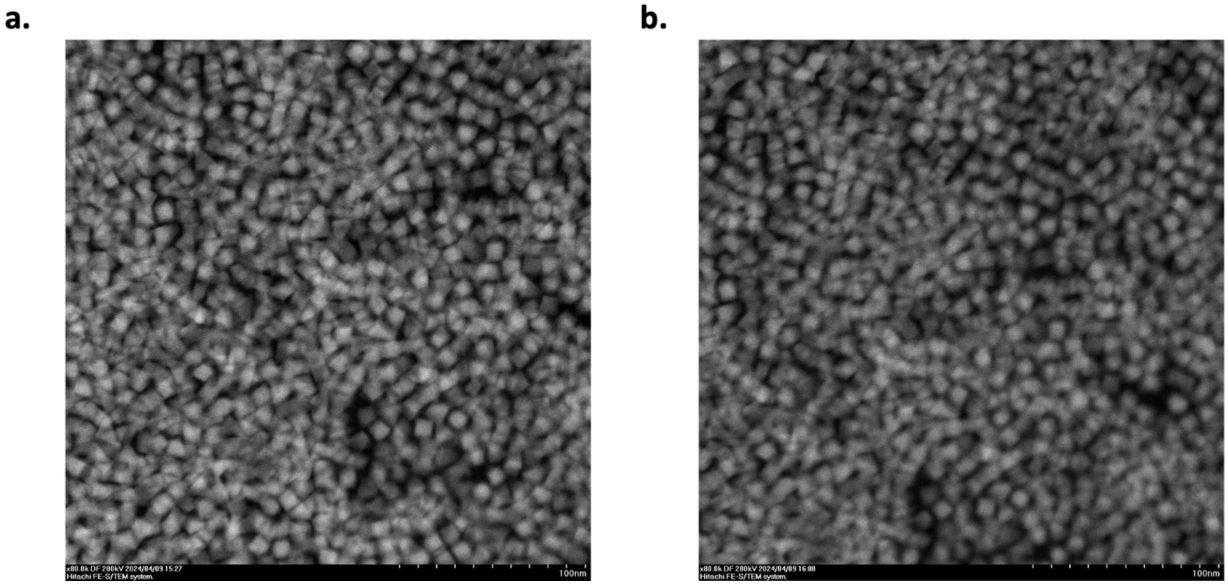

Fig. S22. Influence of H_2 on ZnTeSe/ZnSe/ZnS QDs

a. Pristine blue ZnTeSe/ZnSe/ZnS QD thin film in Area 3. **b.** Hydrogen doped film followed by an immediate rapid beam irradiation (with electron beam dose of $1.59 \times 10^4 \frac{e^-}{\text{Å}^2}$) to capture the image of the thin film in the same region as **a**. QD film coarsening is not observed (same images as presented in Fig. 5c).

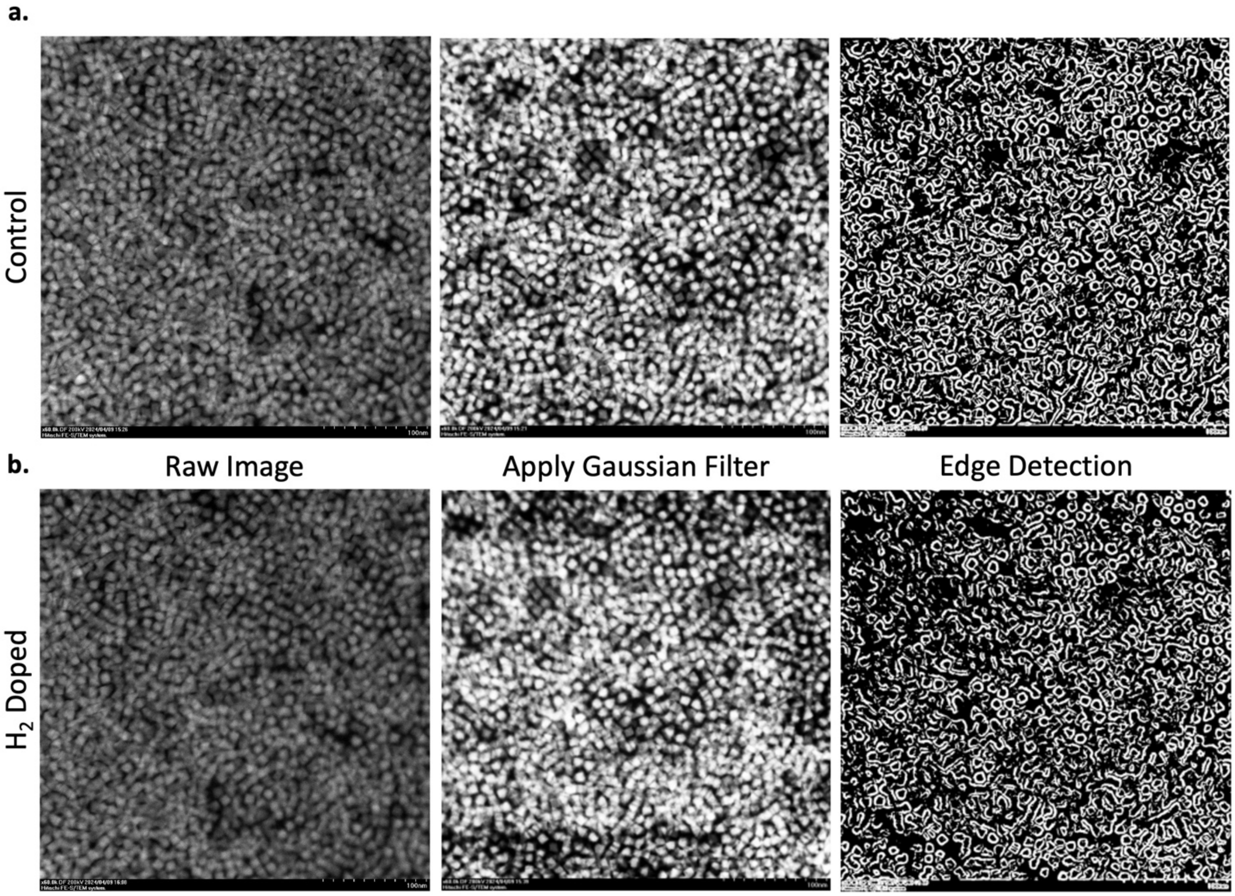

Fig. S23. ZnTeSe/ZnSe/ZnS QD Film Morphology Comparison ---- Computational Method

Image processing of ZnTeSe/ZnSe/ZnS QD thin film HR-TEM images to analyze the sizes of constituent QDs. Images of films of **a.** pristine QDs and **b.** hydrogen doped QDs (from Area 3, defined in Fig. S20a) are processed by applying gaussian filter followed by an edge detection algorithm. QD sizes are then determined by pixel-wise identification by the algorithm.

Demo Codes

Computational Algorithm in TEM Analysis

```
import numpy as np
import matplotlib.pyplot as plt
import torch
import pandas as pd
import cv2
import matplotlib.image as mpimg
from skimage import data
from skimage.color import rgb2gray
from PIL import Image

### Red
red_driven_raw = cv2.imread('Red_LT50_Resin.png') # Red_Driven
red_undriven_raw = cv2.imread('Red_Pristine_Resin.png') # Red_Undriven
plt.imshow(red_driven_raw)
plt.show()
plt.imshow(red_undriven_raw)
plt.show()

# Scale bar Length Determination
start_num = 24
end_num = 324
plt.imshow(red_driven_raw[1800:-1, start_num:end_num])
plt.show()
print(red_driven_raw[1800:-1, start_num:end_num].shape)

# Image Formatting
red_driven = rgb2gray(red_driven_raw)
print(red_driven.shape)
colume = red_driven[:,10]
print(colume.shape)

### Plot Red
colume_1 = []
pixel_num = 2046
spectrum = []
for i in range (2047):
    colume_1 = red_driven[:,i]
    spectrum.append(red_driven[:,i])
    plt.plot(colume_1[0:1930]*255,x_scale[0:1930], color = 'red', linewidth=0.5)
avg = np.mean(spectrum, axis = 0)
plt.plot(avg[0:1930]*255,x_scale[0:1930], '-', color = 'blue')
plt.axis('off')

## Get Layer Thickness
```

```python
colume_2 = []
final_index = []
region_1_mid = 0
region_2_mid = 0
region_3_mid = 0
region_4_mid = 0
length_1 = []
length_2 = []
length_3 = []

for z in range (2048):
    colume_2 = red_driven[:,z]

    region_1 = colume_2[220:370]
    region_2 = colume_2[770:970]
    region_3 = colume_2[1030:1200]
    region_4 = colume_2[1750:1910]

    max_index_1 = np.argmax(region_1)+220
    min_index_1 = np.argmin(region_1)+220
    region_1_mid = int((min_index_1 - max_index_1)/2 + max_index_1)

    max_index_2 = np.argmax(region_2)+770
    min_index_2 = np.argmin(region_2)+770
    region_2_mid = int((min_index_2 - max_index_2)/2 + max_index_2)

    max_index_3 = np.argmax(region_3)+1030
    min_index_3 = np.argmin(region_3)+1030
    region_3_mid = int((max_index_3 - min_index_3)/2 + min_index_3)

    max_index_4 = np.argmax(region_4)+1750
    min_index_4 = np.argmin(region_4)+1750
    region_4_mid = int((max_index_4 - min_index_4)/2 + min_index_4)

    length_1.append(region_2_mid - region_1_mid + 1)
    length_2.append(region_3_mid - region_2_mid + 1)
    length_3.append(region_4_mid - region_3_mid + 1)

avg_1 = np.average(length_1)

avg_2 = np.average(length_2)

avg_3 = np.average(length_3)


print('ZnMgO thickness scale is: ', avg_1)
print('NPs thickness scale is: ', avg_2)
print('Organics thickness scale is: ', avg_3)
```

```
print('ZnMgO thickness  is: ', avg_1*20/300)
print('NPs thickness  is: ', avg_2*20/300)
print('Organic thickness  is: ', avg_3*20/300)

plt.imshow(red_driven_raw)
plt.show()

print(np.shape(spectrum))
```

Title for Supplementary Movie 1:

Evolution of ZnMgO NPs under e-beam

Title for Supplementary Movie 2:

Evolution of BQD under e-beam
